# High-Density Lipoprotein Signaling via Sphingosine-1-Phosphate Receptors Safeguards Spontaneously Hypertensive Rats against Myocardial Ischemia/Reperfusion Injury

**DOI:** 10.3390/pharmaceutics16040497

**Published:** 2024-04-03

**Authors:** Aishah Al-Jarallah, Fawzi A. Babiker

**Affiliations:** 1 Department of Biochemistry, College of Medicine, Kuwait University, Safat 13060, Kuwait; 2 Department of Physiology, College of Medicine, Kuwait University, Safat 13060, Kuwait; fawzi.babiker@ku.edu.kw

**Keywords:** hypertension, ischemia/reperfusion injury, SR-BI, HDL, S1PRs

## Abstract

Background: High-density lipoprotein (HDL) protects against ischemia/reperfusion (I/R) injury via signaling through scavenger-receptor class B type-I (SR-BI) and sphingosine-1-phosphate receptors (S1PRs). We recently reported that HDL protects the hearts of spontaneously hypertensive rats (SHRs) against I/R injury in an SR-BI-dependent manner. Objective: In this study, we examined the role of S1PRs in HDL-induced protection against myocardial I/R injury in hypertensive rats. Methods: Hearts from Wistar Kyoto rats (WKYs) and SHRs were subjected to I/R injury using a modified Langendorff system. The hearts were treated with or without HDL in the presence or absence of a receptor- or kinase-specific antagonist. Cardiac hemodynamics and infarct size were measured. Target proteins were analyzed by immunoblotting and ELISA, and nitrite levels were measured using Greis reagent. Results: HDL protected the hearts of WKYs and SHRs against I/R injury. HDL, however, was more protective in WKYs. HDL protection in SHRs required lipid uptake via SR-BI and S1PR1 and S1PR3 but not S1PR2. The hearts from SHRs expressed significantly lower levels of S1PR3 than the hearts from WKYs. HDL differentially activated mediators of the SAFE and RISK pathways in WKYs and SHRs and resulted in nitric oxide generation. Blockage of these pathways abrogated HDL effects. Conclusions: HDL protects against myocardial I/R injury in normotensive and hypertensive rats, albeit to varying degrees. HDL protection in hearts from hypertensive rodents involved SR-BI-mediated lipid uptake coupled with signaling through S1PR1 and S1PR3. The extent of HDL-induced cardiac protection is directly proportional to S1PR3 expression levels. Mechanistically, the safeguarding effects of HDL involved activation of the SAFE and RISK pathways and the generation of nitric oxide.

## 1. Introduction

Hypertension, a major contributor to early death, remains a key risk factor for cardiac problems, accounting for almost half of coronary heart disease cases [1]. Strategies focusing on blood pressure reduction have demonstrated efficacy in mitigating blood pressure-linked fatalities [2]. Clinical trials have shown that people at high risk of heart problems or those with existing heart disease benefit from blood pressure-lowering treatments, regardless of whether they have normal or high blood pressure [2,3].

A strong inverse relationship exists between the levels of high-density lipoprotein (HDL) and the risk of CHD [4]. Both low HDL cholesterol (HDL-C) levels and hypertension are markers of metabolic syndrome and predictive indicators for acute myocardial infarction [5]. HDL protects against atherosclerotic cardiovascular disease via different mechanisms [6]. The relationship between HDL and hypertension is, however, less clearly understood. Elevated blood pressure nullifies the protective effects of HDL against CHD and stroke [3]. Hypertension and anti-hypertensive drugs alter HDL function and composition [7,8,9].

HDL administration in vivo protects against ischemia/reperfusion (I/R) injury and improves coronary hemodynamics and cardiac functions in isolated rodent hearts [10]. In addition, HDL infusion 30 min prior to the ligation of the left anterior descending (LAD) coronary artery followed by blood restoration for 24 h reduces infarct size, cardiomyocytes necrosis, and inflammation [11]. Reconstituted HDL (rHDL), however, has been shown to be 50% less efficient than native plasma-derived HDL in protecting against myocardial I/R injury [12]. Reconstitution of HDL particles in the presence of sphingosine-1-phosphate (S1P) remarkably improves the cardioprotective properties of HDL ex vivo and in vivo [13].

HDL manifests its protective effects by acting on a range of cell types including cardiomyocytes, endothelial cells, and leukocytes. HDL-induced cardiac protection involves simultaneous inhibition of the damaging effects of I/R injury and activation of internal protective responses. HDL inhibits the damaging effects of I/R injury by scavenging cardiac tumor necrosis factor alpha (TNF-α) [10,12], preventing its damaging effects on cardiomyocytes [14]. HDL induces activation of internal protective responses involved in the activation of TNF-α and signal transducer and activator of transcription-3 (STAT-3) components of the survivor activating factor enhancement (SAFE) pathway and subsequent inhibition of mitochondrial permeability transition pore (mPTP) opening [15]. In addition, HDL administration stimulates the release of vasoactive nitric oxide [11] and cardioprotective prostaglandins [10]. HDL-induced nitric oxide production is mediated by sphingosine-1-phosphate receptor 3 (S1PR3) and involves signaling via phosphatidyl inositol triphosphate kinase (PI3K)/Akt components of the reperfusion injury salvage (RISK) pathway [11].

Hearts from hypertensive rodents exhibit resistance to protection conferred by ischemic postconditioning [16], erythropoietin [17], helium [18], and captopril [19]. We have recently shown that hearts from spontaneously hypertensive rats are, however, responsive to HDL-mediated cardiac protection. HDL-induced protection is dependent on SR-BI and is proportional to SR-BI expression levels in these rats [20]. In addition to SR-BI, S1PR3 mediates the cardioprotective effects of HDL in normotensive rodents [11]. S1PR1, S1PP2, and S1PR3 are expressed in the myocardium [21], and SR-BI interacts with S1PR1 [22]. The role of S1PRs in HDL-mediated cardiac protection from I/R injury in hypertension has, however, not been previously addressed. In this study, we tested the role of S1PR-mediated signaling in HDL-induced cardiac protection in hypertensive rats and compared it to HDL-induced protection in normotensive controls. We demonstrated that HDL protects hearts of hypertensive rats in a dose-dependent manner. HDL retained its protective effects whether administered pre- or post-ischemic injury. In addition, HDL-induced cardiac protection in hypertensive rats required SR-BI-mediated lipid uptake and signaling via S1PR1 and S1PR3. S1PR2, however, appeared to be dispensable. HDL-induced protection was attenuated in hearts from hypertensive rats compared to normotensive controls. Interestingly, the extent of HDL-mediated protection was proportional to the expression level of S1PR3. Moreover, HDL-mediated protection required mediators of the RISK (Akt, ERK1/2) and SAFE (STAT-3) pro-survival pathways and involved the generation of nitric oxide.

## 2. Materials and Methods

### 2.1. Animals and Instrumentation

Randomized twelve- to thirteen-week-old male normotensive Wistar Kyoto rats (WKYs) and spontaneously hypertensive rats (SHRs) were used in this study. Systolic blood pressure values of <120 and >160 mmHg were used as cutoffs for normotensive and hypertensive rats, respectively. Hemodynamics data were obtained from a sample size of 8–12 animals per group, while immunoblotting analysis was conducted using 4–6 animals per group, a sample size selected based on our previous studies [20]. The animals were housed according to globally recognized standards at the Animal Resource Center within the Faculty of Medicine, Kuwait University, and were provided unrestricted access to both food and water [20]. All conducted procedures were approved by the Health Sciences Research Ethics Committee. Briefly, the isolated hearts were retrogradely perfused using freshly prepared Krebs-Hensleit buffer (117.86 mM NaCl, 5.59 mM KCl, 2.40 mM CaCl_2_.2H_2_O, 20.00 mM NaHCO_3_, 1.19 mM KH_2_PO_4_, 1.20 mM MgCl_2_.6H_2_O, and 12.11 mM glucose), pH 7.35 to 7.45 at 37.0 ± 0.5 °C, and gassed with a mixture of CO_2_ (5%) and O_2_ (95%). To induce regional ischemia, the left anterior descending (LAD) coronary artery was occluded for a duration of 30 min. Throughout all experimental protocols, preload was maintained at a constant level of 6 mmHg under controlled basal conditions, and the perfusion pressure (PP) was set to 50 mmHg. The perfusion pressure was measured downstream from an aortic cannula branch, utilizing a Statham pressure transducer (P23 Db). A constant perfusion pressure was achieved electronically using the Module PPCM type 671 (Hugo Sachs Elektronik-Harvard Apparatus GmbH, Grünstraße, Germany), an effective system that allows precise PP adjustment within a range of 5 mmHg to 150 mmHg, with an accuracy level of ±1 mmHg.

### 2.2. Study Protocol and Study Groups

**Figure 1:**
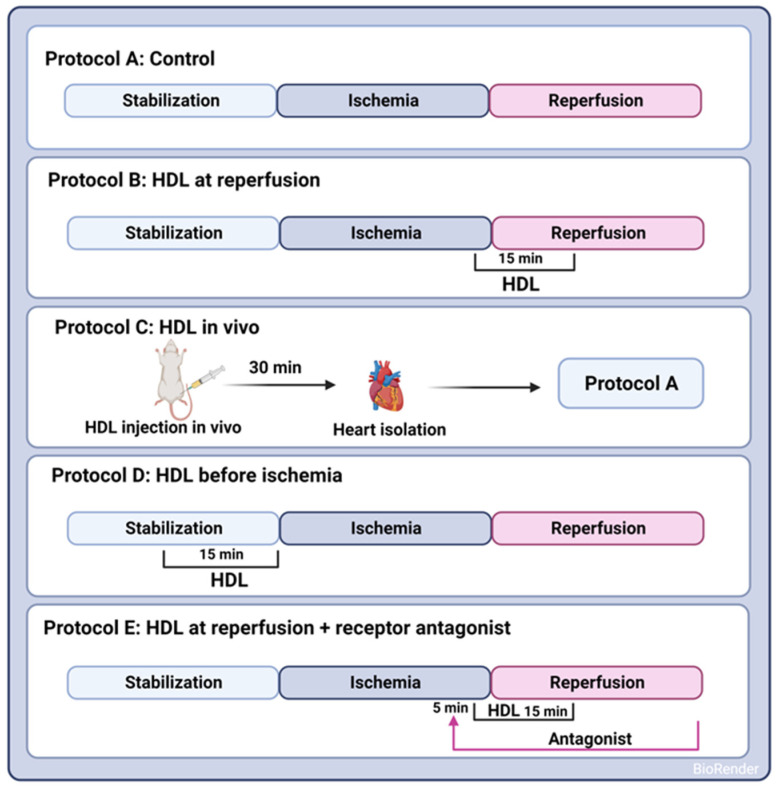
Experimental protocols used to examine the HDL effects on I/R injury in SHRs. In Protocols A–E, hearts were stabilized (30 min). Hearts were then subjected to ischemic injury (30 min) without further additions (Protocol A); HDL was administered either at reperfusion (Protocol B), in vivo by intravenous injection 30 min prior to sacrifice (Protocol C), or infused ex vivo prior to ligation (Protocol D) or 5 min after the addition of receptor specific antagonist (Protocol E). Hearts (A–E) were then perfused for 30 min.

A uniform procedure involved subjecting all hearts to a 30 min ischemic phase, induced by occluding the LAD coronary artery. This occlusion was achieved by encircling the LAD coronary artery with a snare positioned approximately 0.5 cm below the atrioventricular groove. To guarantee complete occlusion of the coronary artery, a small rigid plastic tube was placed between the heart and the snare. After the ischemic interval, a 30 min reperfusion period followed for all hearts. Control hearts were perfused without any additions to the perfusion buffer (protocol A, Figure 1). HDL (100 µg or 400 µg, Lee Biosolutions, Maryland Heights, MO, USA), lipid-free apo-AI (400 µg, Sigma Aldrich, Munich, Germany), or HDL3 (100 µg, Biorbyt, Cambridge, UK) in a total volume of 150 mL of reperfusion buffer was administered at the last five minutes of ischemia and continued for the first ten minutes of the reperfusion period (protocol B, Figure 1). To assess the effect of pretreatment with HDL on I/R injury, HDL (400 µg) was infused intravenously two hours prior to sacrifice (protocol C, Figure 1) or 15 min prior to ischemia (protocol D, Figure 1). The selected dose of HDL was based on previous data generated by us [20] and others [11]. To evaluate the effects of receptor-mediated signaling, hearts were treated with HDL in the presence or absence of BLT-1 (inhibitor of SR-BI-mediated lipid uptake, 1 µM, Sigma Aldrich, Munich, Germany), W146 (S1PR1 antagonist, 10 µM, Cayman Chemical Ann Arbor, MI, USA), JTE-013 (S1PR2 antagonist, 1 µM, Cayman Chemical, Ann Arbor, MI, USA), or CAY-10444 (S1PR3 antagonist, 10 µM, Cayman Chemical, Ann Arbor, MI, USA). To test the role of intracellular signaling pathways, hearts were treated with HDL in the presence or absence of LY294002 (PI3K antagonist, 10 µM, Cayman Chemical, Ann Arbor, MI, USA), PD98059 (MEK1 agonist, 10 µM, Cell Signaling Technology, Danvers, MA, USA), Stattic (STAT-3 antagonist, 20 µM, Merk Millipore, Espoo, Finland), or SP600125 (JNK antagonist, 20 µM, Cayman Chemical, Ann Arbor, MI, USA). The antagonist was infused 5 min prior to the addition of HDL (400 µg) and continued in the presence of HDL for the first ten minutes of reperfusion (protocol E, Figure 1).

### 2.3. Data Collection and Processing

To gauge left ventricular (LV) function, we assessed parameters including LV end diastolic pressure (LVEDP) and LV maximum developed pressure (DPmax). Additionally, coronary vascular dynamics were explored through the evaluation of coronary flow (CF) and coronary vascular resistance (CVR) [20]. In brief, a water-filled latex balloon was carefully placed within the LV cavity. This balloon was linked to both a pressure transducer and a DC bridge amplifier (DC-BA) featuring an integrated pressure module (DC-BA type 660, Hugo-Sachs Electronik, Grünstraße, Germany). This configuration facilitated real-time monitoring of DPmax, seamlessly interfaced with a personal computer. The LV developed pressure was computed from online acquisition of LV end systolic pressure (LVESP) through a max–min module (Number MMM type 668, Hugo Sachs Elektronik-Harvard Apparatus GmbH, Grünstraße, Germany). This module performed a mathematical transformation of the DC bridge amplifier’s output, deriving DPmax by subtracting LVEDP from LVESP. Coronary flow, however, was tracked with an electromagnetic flow probe attached to the aortic cannula’s inflow, which was linked to a personal computer for continuous measurement of coronary flow in mL/min. To ensure data accuracy, we used software tailored specifically for this purpose, verified by manual collection of coronary effluent at specific intervals. The recording of CVR and hemodynamic data occurred in 10 s intervals, facilitated by an online data acquisition program (Isoheart software V 1.524-S, Hugo-Sachs Electronik, Grünstraße, Germany). At the end of each experiment, hearts were frozen in liquid nitrogen and stored at −80 °C for further analysis.

### 2.4. Measurements of Infarct Size by Triphenyltetrazolium Chloride Staining

Heart specimens were collected after reperfusion and stored at −20 °C overnight. Each heart was transversely sliced into 4–6 sections from the apex to the base. These sections were then immersed in triphenyltetrazolium chloride (TTC) solution (1%) in isotonic phosphate buffer (pH 7.40) for 24 h before being fixed in 4 percent formaldehyde. The infarct size was assessed blindly using ImageJ software (version v1.54d; National Institute of Health, Bethesda, MD, USA; n = 3/group). The viable areas (red) and the necrotic infarcted regions (pale, non-stained) were manually detected for each section. The infarct area was expressed as a percentage of the total LV area.

### 2.5. Analysis of Serum Lipids

Serum samples were collected and enzymatically assayed for total cholesterol (TC), HDL, non-HDL cholesterol (Abcam, Cambridge, UK), unesterified cholesterol (FC), and triglycerides (TG, Wako Diagnostics, Richmond, VA, USA) as indicated in the manufacturer data sheet. HDL subpopulations were analyzed using the Lipoprint System (Quantimatrix, Redondo Beach, CA, USA). Briefly, serum samples (25 µL) were mixed with HDL liporprint loading gel (300 µL). The gel was then photopolymerized for 30 min and run at 500 V for 50 min. Finally, the gel was scanned and analyzed using the Lipoprint System (Lipoware LW03-v.16-134), and the proportion of HDL subpopulations was determined and compared between serum samples from WKYs and SHRs. S1P levels were measured in heart homogenates from WKYs and SHRs by ELISA (MyBiosource, San Diego, CA, USA) following the manufacturer’s instructions.

### 2.6. Immunoblotting and Enzyme-Linked Immunosorbent Assay

Membrane preparations from WKY and SHR hearts were used to determine the expression levels of cell surface receptors including S1PR1, S1PR2, and S1PR3. Whole hearts were homogenized on ice for 3 min in 20 mM Tris-HCl, pH 7.5, containing 2 mM MgCl_2_, 0.25 M sucrose, and 1× protease inhibitors (Sigma Aldrich, Munich, Germany). Homogenates were centrifuged at 3000× *g* for 10 min at 4 °C, and supernatants were subjected to another centrifugation step at 100,000× *g* for 1 h at 4 °C. The pellet was suspended in 50 mM Tris-HCl, pH 7.5, containing a 1× protease inhibitor cocktail (Sigma Aldrich, Munich, Germany) and 0.1% sodium dodecyl sulfate. The expression levels of signaling proteins were detected in total homogenates from WKY and SHR hearts subjected to I/R injury (protocol B, Figure 1). Briefly, the snap-frozen hearts were homogenized in 0.2× PBS, 0.1% triton-×100, 1× phosphatase inhibitor, and 1× protease inhibitor cocktail then centrifuged at 10,956× *g* in a bench-top minicentrifuge for 10 min at 4 °C. Protein concentrations were measured using the BCA-protein determination kit (Themo Scientific, Ottawa, ON, Canada), and samples were stored at −80 °C for further analysis. After boiling, samples (50 μg protein) were subjected to SDS-PAGE, and PVDF membranes were immunoblotted with rabbit anti-S1PR1 (Cayman Chemical, MI, USA), rabbit anti-S1PR2 (Sigma Aldrich, Munich, Germany), rabbit anti-S1PR3 (Cayman Chemicals, MI, USA), rabbit anti-phospho- and total Akt, ERK1/2, STAT-3, JNK1/2 (Cell Signaling Technology, Danvers, MA, USA), and rabbit anti-GAPDH (Cell Signaling Technology, Danvers, MA, USA). This was followed by HRP-conjugated donkey anti-rabbit antibody (Jackson ImmunoResearch, West Grove, PA, USA). Bands were detected using Super Signal Western Pico Chemiluminescence Substrate (Thermo Scientific, Ottawa, ON, Canada) and quantified using the Bio-Rad gel doc RX System (Bio-Rad, Hercules, CA, USA). S1PR1 (Assay Genie, Dublin, Ireland), S1PR2 (Abcam, Cambridge, UK), and S1PR3 (Aviva system, San Diego, CA, USA) levels in membrane preparations from WKYs and SHRs were also measured by enzyme-linked immunosorbent assay (ELISA) according to the manufacturer’s instructions and were expressed relative to total protein concentrations.

### 2.7. Measurements of Nitric Oxide

Nitrite contents as a measure of nitric oxide levels were determined in total heart homogenates from WKYs and SHRs using Griess reagent. Briefly, 1:2 volume of the samples or sodium nitrite standards were added to 1% Griess reagent, incubated for 20 min at room temperature, and then quantified spectrophotometrically at 540 nm.

### 2.8. Statistical Analysis

Data normality was tested using the Shapiro–Wilk test. The Student *t*-test was used to compare two groups that followed a normal distribution, while the Mann–Whitney U-test was used to compare two groups that failed to follow a normal distribution. One-way analysis of variance (ANOVA) followed by Dunnett’s test was used to compare more than two groups with a single variable. Two-way ANOVA followed by the Tukey test was used to determine differences between multiple groups with two variables. *p* < 0.05 was considered statistically significant. The data are represented as the mean ± standard error of the mean (SEM).

## 3. Results

### 3.1. SHRs Displayed Elevated Blood Pressure and Dysregulated Lipid Metabolism

SHRs demonstrated significantly (*p* < 0.01) elevated SBP and DBP levels and increased heart rate, blood flow, and volume relative to WKYs (Supplementary Table S1). SHRs had significantly (*p* < 0.05) lower body weights and significantly (*p* < 0.001) higher heart to body weight ratios compared to WKYs (Supplementary Table S1). Moreover, SHRs had significantly (*p* < 0.001) lower levels of total cholesterol and triglycerides (Supplementary Figure S1A,B) and significantly (*p* < 0.05) higher levels of non-HDL cholesterol and free cholesterol (Supplementary Figure S1C,D) relative to WKYs. HDL from SHRs demonstrated altered composition and subpopulation profiles (Supplementary Figure S1E–H). HDL cholesterol contents were significantly (*p* < 0.001) reduced, while the HDL-S1P contents were significantly (*p* < 0.05) increased in SHRs compared to WKYs (Supplementary Figure S1E,G). HDL free cholesterol levels, however, were not significantly different between the two groups (Supplementary Figure S1F). Furthermore, the proportion of large and medium HDL particles was significantly (*p* < 0.05) reduced and increased in SHRs, respectively, relative to WKYs (Supplementary Figure S1H). HDL from SHRs contained a higher level of small HDL particles, yet the difference between the two groups did not reach statistical significance (Supplementary Figure S1H). Collectively, these data suggest altered serum lipids and lipoprotein profiles in hypertensive rats relative to normotensive controls. Increased non-HDL cholesterol, reduced HDL cholesterol, and altered HDL composition and subpopulations may contribute to the hypertension-induced cardiovascular complications observed in SHRs.

### 3.2. HDL Induces Cardiac Protection in WKYs and SHRs in a Dose-Dependent Manner

**Figure 2:**
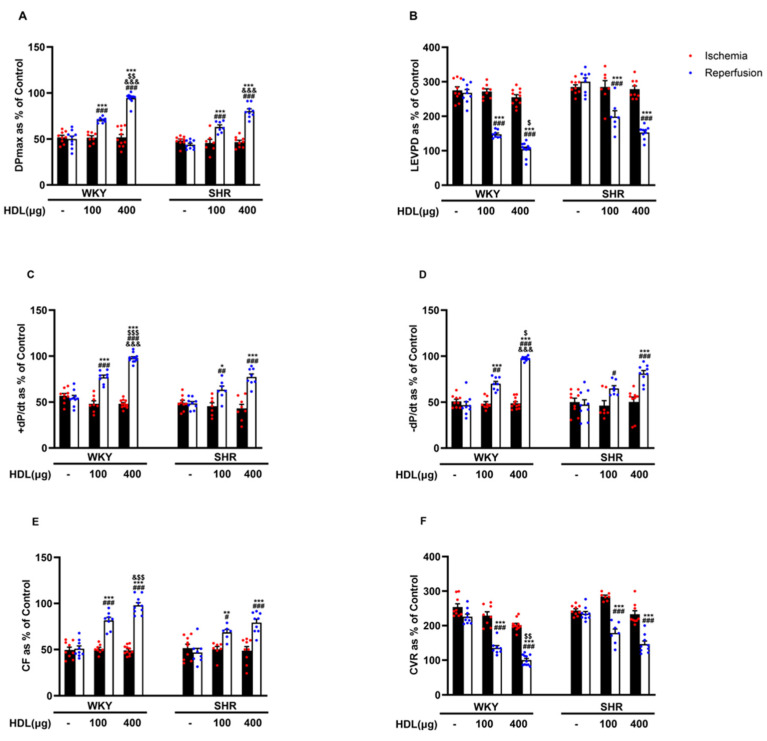
HDL induces cardiac protection in WKYs and SHRs in a dose-dependent manner. Post-ischemic recovery parameters of the heart functions including DPmax (**A**), LVEDP (**B**), cardiac contractility (**D**,**E**), CF (**C**), and CVR (**F**). The data were computed at 30 min of HDL (100 and 400 µg) infused at reperfusion and expressed as the mean ± SEM. DPmax, maximum developed pressure; LVEDP, left ventricular end diastolic pressure; CF, coronary flow; CVR, coronary vascular resistance. * *p* < 0.05, ** *p* < 0.01, and *** *p* < 0.001 vs. untreated control; ^#^ *p* < 0.05, ^##^ *p* < 0.01, and ^###^ *p* < 0.001 vs. ischemia; ^$^ *p* < 0.05, ^$$^ *p* < 0.01, and ^$$$^ *p* < 0.001 vs. SHR+HDL (400 µg); and ^&^ *p* < 0.05, and ^&&&^ *p* < 0.001 vs. HDL (100 µg) of the same genotype.

We tested the effect of HDL (100 µg and 400 µg) infused at the beginning of reperfusion on I/R-induced cardiac injury in normotensive and hypertensive rats (Figure 2, Supplementary Figure S2). HDL (100 µg and 400 µg) significantly (*p* < 0.05) improved LV function, DPmax and LVEDP (Figure 2A,B), LV contractility, +dp/dt and −dp/dt (Figure 2C,D), and coronary–vascular dynamics, CF, and CVR (Figure 2E,F) with enhanced protection observed in WKYs. In addition, HDL significantly (*p* < 0.05) reduced the infarct size in hearts from WKYs and SHRs (Supplementary Figure S2).

Moreover, we tested if the cardioprotective effects of HDL can be replicated by HDL major apo-lipoprotein, apo-AI, or small HDL particles, HDL3 (Supplementary Figure S3). Infusion of lipid-free apo-AI or HDL3 at reperfusion did not protect the hearts of normotensive or hypertensive rats against myocardial I/R injury (Supplementary Figure S3), suggesting that lipid-free apo-AI and small HDL particles are not sufficient to protect against I/R injury. This possibly implicates the requirement for additional HDL components and/or subpopulations in the protective actions of HDL. We further tested if pretreatment with HDL is protective against myocardial I/R injury in WKYs and SHRs. HDL (400 µg) was infused ex vivo, 15 min prior to ligation, or intravenously, 30 min prior to sacrifice (Supplementary Figure S4). Pretreatment with HDL protected WKYs and SHRs against I/R injury as indicated by the significant (*p* < 0.05) improvements in LV function (Supplementary Figure S4A,B), LV contractility (Supplementary Figure S4C,D), and coronary–vascular dynamics (Supplementary Figure S4E,F). Together, these data suggest that both pretreatment and posttreatment with HDL protect against myocardial I/R injury in normotensive and hypertensive rats.

### 3.3. HDL-Induced Cardiac Protection Requires SR-BI-Mediated Lipid Uptake

**Figure 3:**
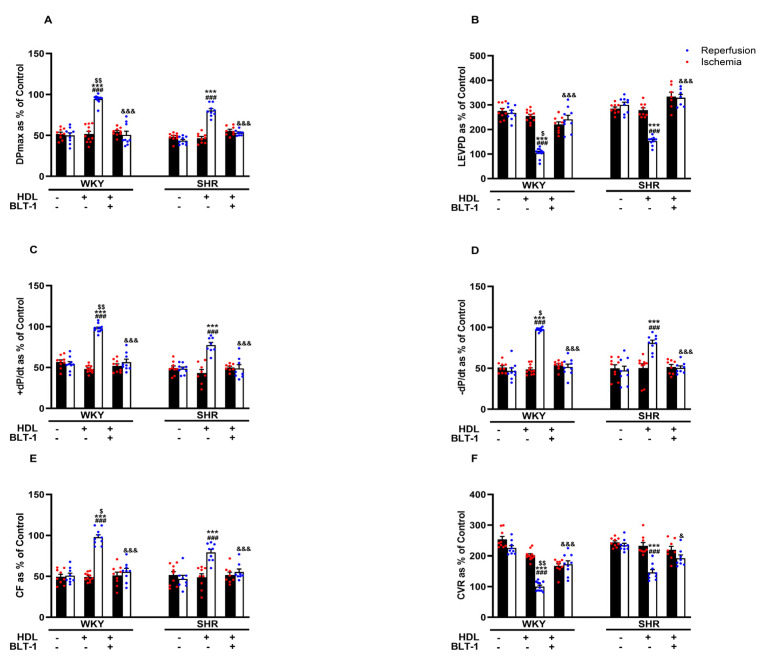
HDL-induced cardiac protection requires SR-BI-mediated lipid uptake. Post-ischemic recovery parameters of the heart function including DPmax (**A**), LVEDP (**B**), cardiac contractility (**C**,**D**), and coronary hemodynamics (**E**,**F**) were monitored. The data were computed with no addition control, with HDL (400 μg) administered at reperfusion in the presence or absence of BLT-1 (1 μM). DPmax, maximum developed pressure; LVEDP, left ventricular end diastolic pressure; CF, coronary flow; CVR, coronary vascular resistance; BLT-1, blocker of lipid transport-1. The data are the mean ± SEM. *** *p* < 0.001 vs. untreated control; ^###^ *p* < 0.001 vs. ischemia; ^$^ *p* < 0.05, ^$$^ *p* < 0.01, vs. SHR+HDL (400 µg); and ^&^ *p* < 0.05, and ^&&&^ *p* < 0.001 vs. HDL (400 µg) of the same genotype.

Reduced HDL-mediated protection against I/R injury in SHRs (Figure 2) may suggest attenuated HDL-mediated signaling in these rats. We have previously reported reduced SR-BI expression levels in membrane fractions from hypertensive rats and the requirement of HDL binding to SR-BI in HDL-mediated protection against myocardial I/R injury [20]. In this study, we investigated the involvement of SR-BI-mediated lipid update in HDL-induced cardiac protection using a blocker of lipid uptake, BLT-1. Blockage of SR-BI-mediated lipid uptake abrogated the protective effects of HDL in hearts from WKYs and SHRs (Figure 3A–F, Supplementary Figure S2), suggesting the requirement of SR-BI-mediated lipid uptake in HDL-induced cardiac protection in normotensive and hypertensive rats.

### 3.4. HDL-Induced Cardiac Protection Is Mediated by S1PR1 and S1PR3

**Figure 4:**
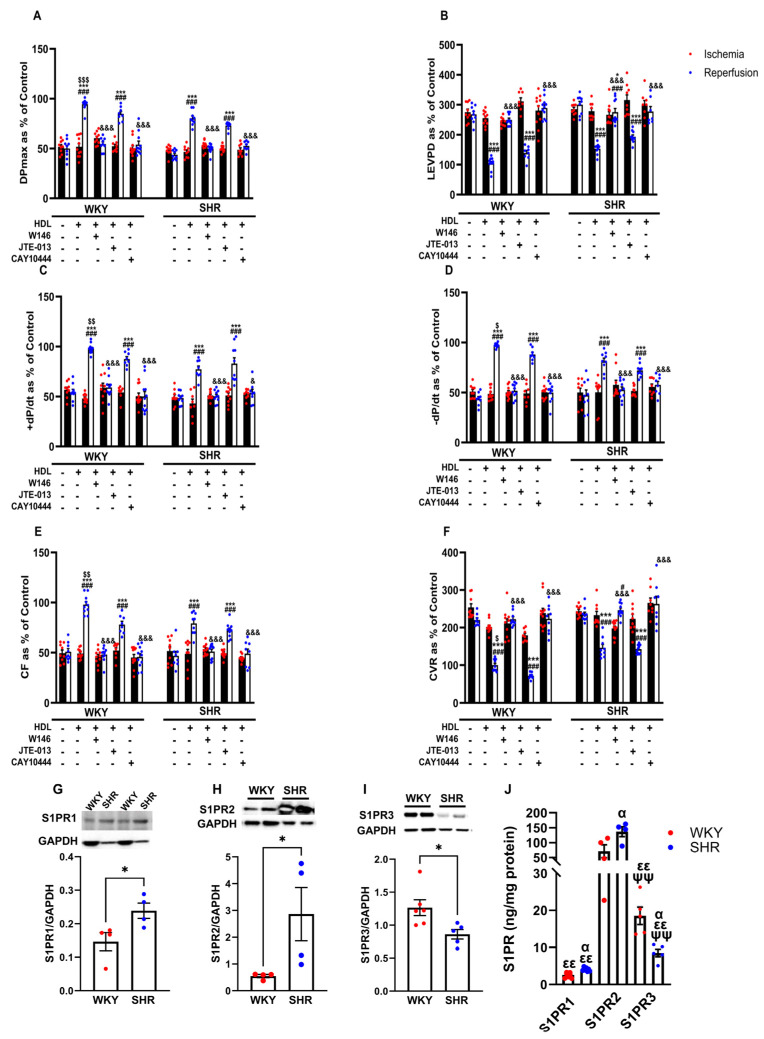
Role of S1PRs in HDL-induced cardiac protection. Post-ischemic recovery parameters of the heart function including DPmax (**A**), LVEDP (**B**), cardiac contractility (**C**,**D**), CF (**E**), and CVR (**F**) were monitored. The data were computed with no addition of HDL, with HDL (400 µg) at reperfusion in the presence or absence of S1PR2 antagonist (W146, 2 µM), S1PR2 antagonist (JTE-013, 1 µM), or S1PR3 antagonist (CAY10444, 10 µM) infused at reperfusion; S1PR1, S1PR2, and S1PR3 levels were detected by immunoblotting (**G**–**I**) or by ELISA (**J**) in hearts from WKYs and SHRs. DPmax, maximum developed pressure; LVEDP, left ventricular end diastolic pressure; CF, coronary flow; CVR, coronary vascular resistance. The data are the mean ± SEM. * *p* < 0.05, and *** *p* < 0.001 vs. untreated control; ^#^ *p* < 0.05, and ^###^ *p* < 0.001 vs. ischemia; ^$^ *p* < 0.05, ^$$^ *p* < 0.01, and ^$$$^ *p* < 0.001 vs. SHR+HDL (400 µg); ^&^ *p* < 0.05, and ^&&&^ *p* < 0.001 vs. HDL (400 µg) of the same genotype; ^α^ *p* < 0.05 vs. WKY; ^εε^ *p* < 0.01 vs. S1PR2 of the same genotype; and ^ψψ^ *p* < 0.01 vs. S1PR1 of the same genotype.

S1PR isoforms 1, 2, and 3 but not 4 and 5 are expressed in the heart [21]. We tested the involvement of S1PR1, 2, and 3 in HDL-induced cardiac protection using receptor-specific antagonists (Figure 4). HDL-induced protection against I/R injury was abolished in the presence of the S1PR1 and S1PR3 antagonists and reduced in the presence of the S1PR2 antagonist in WKYs (Figure 4A–F, Supplementary Figure S2). In SHRs, however, HDL-mediated protection was lost in the presence of the S1PR1 and S1PR3 antagonists, yet was maintained in the presence of the S1PR2 antagonist in SHRs (Figure 4A–F, Supplementary Figure S2). This suggests that HDL-induced protection requires S1PR1, S1PR2, and S1PR3 in normotensive rats. However, HDL action appears to involve S1PR1 and S1PR3 but not S1PR2 in hypertensive rats. We further examined the expression levels of S1PR1, S1PR2, and S1PR3 in membrane preparations from normotensive and hypertensive rats (Figure 4G–J). WKYs expressed significantly (*p* < 0.05) lower levels of S1PR1 and S1PR2 and significantly (*p* < 0.05) higher levels of S1PR3 relative to SHRs (Figure 4G–I). S1PR2 was the most abundantly expressed S1PR isoform relative to S1PR3 and S1PR1 in hearts from WKYs and SHRs (Figure 4J). Moreover, S1PR3 levels were significantly (*p* < 0.01) higher compared to S1PR1 levels in hearts from both genotypes (Figure 4J). Interestingly, HDL-mediated protection against I/R injury correlated negatively with the expression levels of S1PR1 and positively with those of S1PR3 in hearts from WKYs and SHRs (Supplementary Table S2). Furthermore, this correlation was lost in the presence of S1PR1- and S1PR3-specific antagonists (Supplementary Table S2). Cardiac S1PR2 expression, however, did not correlate with the HDL-mediated increase in coronary flow in normotensive and hypertensive rats (Supplementary Table S2). Increased expression of S1PR3 in WKYs relative to SHRs and the significant positive correlation between S1PR3 protein levels and coronary flow could possibly explain the enhanced HDL-mediated cardiac protection in normotensive rats.

### 3.5. HDL-Mediated Cardiac Protection Requires Signaling via the RISK and SAFE Pro-Survival Pathways and Involves the Generation of Nitric Oxide

**Figure 5:**
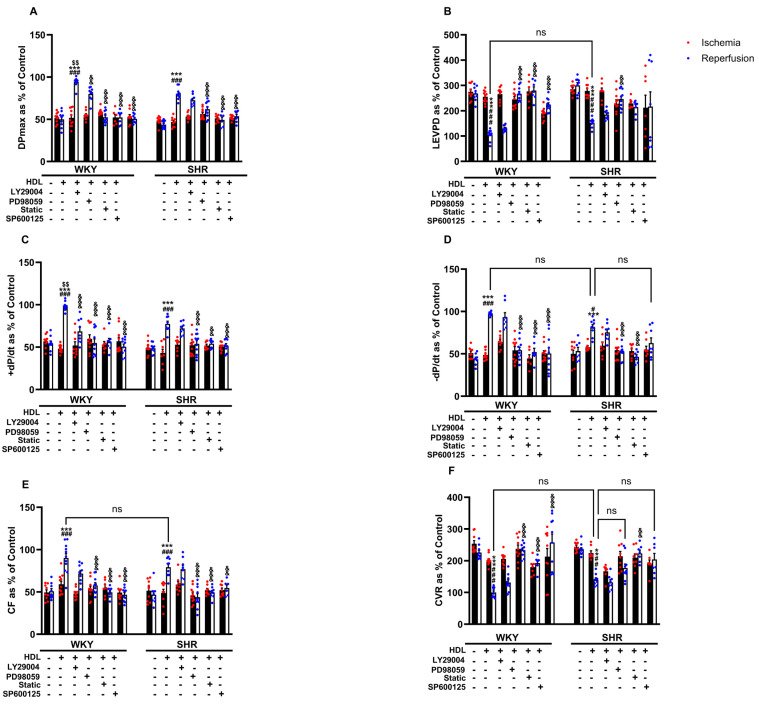
HDL-mediated cardiac protection requires signaling via the RISK and SAFE pro-survival pathways. Post-ischemic recovery parameters of the heart function including DPmax (**A**), LVEDP (**B**), coronary hemodynamics (**C**,**D**), and cardiac contractility (**E**,**F**) were monitored. The data were computed with no addition control, with HDL (400 μg) in the presence or absence of PI3K antagonist (LY294002, 10 μM), MEK antagonist (PD98509, 10 μM), STAT-3 antagonist (Stattic, 20 μM), or JNK antagonist (SP600125, 10 μM). DPmax, maximum developed pressure; LVEDP, left ventricular end diastolic pressure; CF, coronary flow; CVR, coronary vascular resistance. The data are the mean ± SEM. ** *p* < 0.01 and *** *p* < 0.001 vs. untreated control; ^#^ *p* < 0.05, ^##^ *p* < 0.01, and ^###^ *p* < 0.001 vs. ischemia; ^$$^ *p* < 0.01 vs. SHR+HDL (400 µg); and, ^&&^ *p* < 0.01, and ^&&&^ *p* < 0.001 vs. HDL (100 µg) of the same genotype.

The cardioprotective actions of HDL involved the activation of multiple protective intracellular responses including the SAFE and RISK pathways and resulted in the generation of nitric oxide [23]. We therefore tested the involvement of PI3K, STAT-3, ERK1/2, and JNK1/2 in HDL-induced cardiac protection against I/R injury using target-specific antagonists (Figure 5). HDL, in the presence of PI3K antagonist, significantly (*p* < 0.05) improved LV function (DPmax and LVEDP), LV contractility (+dp/dt and −dp/dt), and coronary–vascular dynamics (CF and CVR) relative to ischemia and relative to untreated controls (Figure 5A–F), suggesting that the inhibition of PI3K is not sufficient to block HDL-induced cardiac protection. Alternatively, this may implicate the involvement of PI3K-independent pathways. HDL, in the presence of ERK1/2, STAT-3, or JNK1/2 antagonists, however, did not protect against myocardial I/R injury in normotensive and hypertensive rats (Figure 5A–F), suggesting an indispensable, perhaps a downstream, role of ERK1/2, STAT-3, and JNK1/2 signaling in HDL-induced cardiac protection.

In addition, we tested the effect of HDL on the activation state of mediators of the SAFE and RISK pathways (Supplementary Figure S5). HDL significantly (*p* < 0.05) induced Akt phosphorylation in SHRs but not in WKYs (Supplementary Figure S5A). Total Akt levels were not significantly different between normotensive and hypertensive rats and were not affected by HDL treatment (Supplementary Figure S5B). HDL, however, induced a significant (*p* < 0.05) increase in STAT-3 phosphorylation in WKYs with a trend towards an increase in SHRs (Supplementary Figure S5C). Nonetheless, total-STAT-3 levels were significantly (*p* < 0.05) lower in SHRs compared to WKYs and were not affected by HDL treatment (Supplementary Figure S5D). HDL did not activate ERK1/2 (Supplementary Figure S5E) or JNK1/2 (Supplementary Figure S5G) in WKYs and SHRs at the time point tested. Furthermore, normotensive and hypertensive rats expressed similar levels of total ERK1/2 and JNK1/2 (Supplementary Figure S5F,H). The lack of HDL-induced activation of RISK and SAFE mediators involved in HDL-mediated cardiac protection (Supplementary Figure S5) perhaps suggests the likelihood of an initial activation that might have reverted to baseline after 30 min of reperfusion.

**Figure 6:**
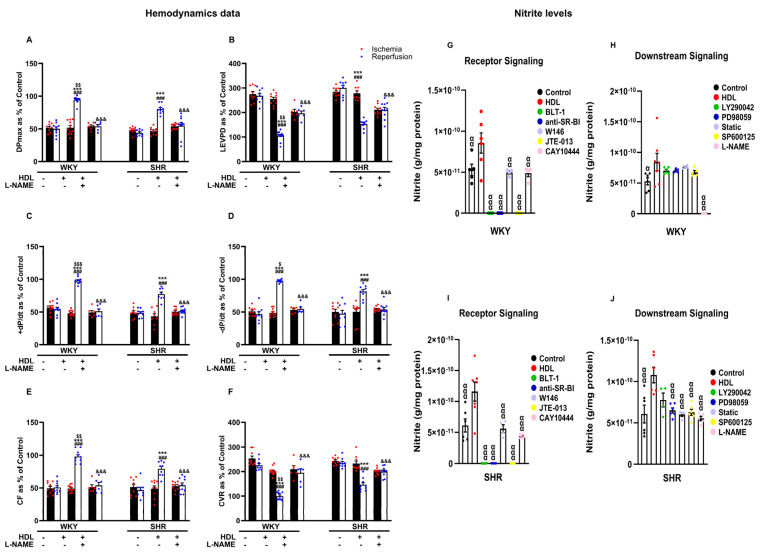
The role of nitric oxide synthase in HDL-mediated cardiac protection against myocardial I/R injury. Post-ischemic recovery parameters of the heart function including DPmax (**A**), LVEDP (**B**), cardiac contractility (**C**,**D**), and coronary hemodynamics (**E**,**F**) were monitored. The data were computed with no addition control, with HDL (400 μg) administered at reperfusion in the presence or absence of nitric oxide synthase inhibitor (L-NAME, 10 μM). Total heart homogenates from WKYs and SHRs treated with or without HDL (400 µg) at reperfusion in the presence or absence of BLT-1 (1 μM), SR-BI blocking antibody (1:100), W146 (2 μM), JTE-013(1 μM), CAY10444 (10 µM), LY294002 (10 μM), PD98509 (10 μM), Stattic (20 μM), SP600125 (10 μM), or L-NAME (10 μM) were assayed for nitric oxide (**G**–**J**). DPmax, maximum developed pressure; LVEDP, left ventricular end diastolic pressure; CF, coronary flow; CVR, coronary vascular resistance. The data are the mean ± SEM. *** *p* < 0.001 vs. untreated control; ^###^ *p* < 0.001 vs. ischemia; ^$^ *p* < 0.05, ^$$^ *p* < 0.01, and ^$$$^ *p* <0.001 vs. SHR+HDL (400 µg); ^&&&^ *p* < 0.001 vs. HDL (400 µg) of the same genotype; and ^α^ *p* < 0.05, ^αα^ *p* < 0.01, and ^ααα^ *p* < 0.001 vs. HDL (400 µg).

Inhibition of nitric oxide synthase with L-NAME attenuated HDL-induced cardiac protection (Figure 6A–F, Supplementary Figure S2), suggesting an essential role of NOS in mediating the cardioprotective effects of HDL in normotensive and hypertensive rats. Finally, we examined the effects of HDL on the production of nitric oxide in WKYs and SHRs (Figure 6G–J). Administration of HDL at reperfusion significantly (*p* < 0.05) increased nitric oxide production in heart homogenates from WKYs (Figure 6G,H) and SHRs (Figure 6I,J), consistent with the observed increase in coronary flow and reduction in coronary resistance in response to HDL (Figure 3E,F). This, however, was completely abrogated (100% reduction) in the presence of BLT-1 or SR-BI blocking antibody, suggesting the requirement of SR-BI-mediated lipid uptake and HDL binding to SR-BI in HDL-induced production of nitric oxide (Figure 6G,I). Blockage of S1PR1 and S1PR3 significantly (*p* < 0.05) reduced HDL-mediated production of nitric oxide in SHRs by 41% and 42% and in WKYs by 45% and 57%, respectively (Figure 6G,I). Blockage of S1PR2, however, completely abolished (100% reduction) HDL-mediated production of nitric oxide in normotensive and hypertensive rats. This suggests the requirement of S1PR1 and S1PR3 in HDL-mediated nitric oxide generation. It further suggests an indispensable role of S1PR2 in HDL-induced nitric oxide production. Blockage of PI3K, however, did not affect HDL-mediated nitric oxide production in WKYs (Figure 6H) and SHRs (Figure 6J). Moreover, blockage of ERK1/2, STAT-3, or JNK1/2 did not reduce HDL-mediated nitric oxide production in WKYs (Figure 6H); nonetheless, it was detrimental in SHRs (Figure 6J), suggesting that blockage of one pathway can be compensated by other pathway(s) in WKYs but not in SHRs. L-NAME treatment completely abrogated (100% reduction) HDL-induced nitric oxide production in WKYs (Figure 6H) and significantly reduced (47% reduction) HDL-mediated nitric oxide production in SHRs (Figure 6J), implicating the presence of NOS-independent HDL-induced generation of nitric oxide in SHRs. Finally, the HDL-mediated increase in coronary flow correlated positively (*p* < 0.05) with the HDL-mediated production of nitric oxide in hearts from normotensive and hypertensive rats, and this correlation was lost in the presence of L-NAME (Supplementary Table S2).

## 4. Discussion

**Figure 7:**
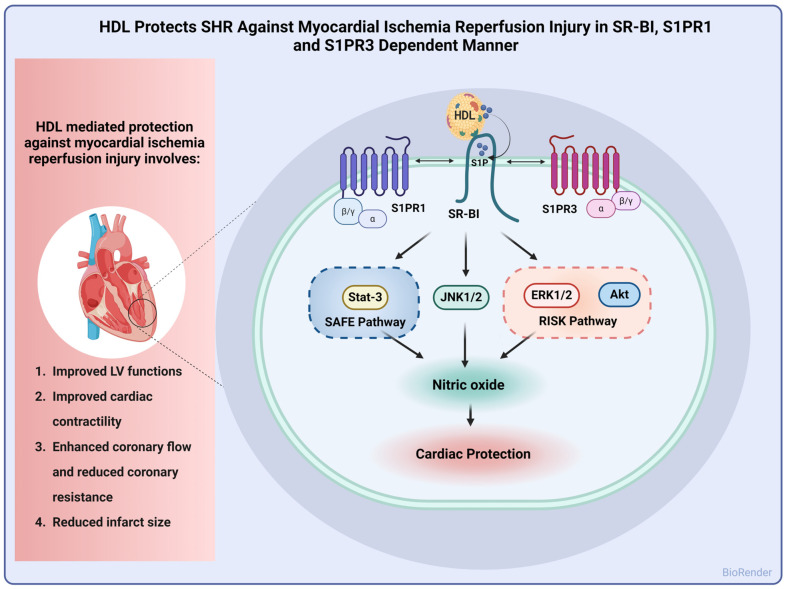
Proposed mechanism of HDL-induced cardiac protection against I/R injury in SHRs. HDL protected hearts of SHRs against I/R injury, as indicated by improved LV functions, cardiac contractility, and hemodynamics and reduced infarct size. Mechanistically, HDL-induced protection involved signaling via SR-BI, S1PR1, and S1PR3 with subsequent activation of the SAFE and RISK pathways and generation of nitric oxide.

In this study, we report alterations in lipid and lipoprotein metabolism in hypertensive rats. HDL particles from SHRs had lower total cholesterol, cholesterol esters, and free cholesterol and higher S1P contents compared to particles from normotensive rats. HDL subspecies were also different between normotensive and hypertensive rats. SHRs had lower contents of large HDL particles and higher levels of medium particles relative to WKYs. In addition, we report a dose-dependent cardioprotective effect of HDL against I/R injury that was reproduced under pre- and posttreatment settings in WKYs and SHRs. HDL-induced cardiac protection required lipid uptake via SR-BI and signaling via S1PR1 and S1PR3. Finally, HDL-induced protection entailed signaling via ERK1/2, STAT-3, and JNK1/2 with concomitant generation of nitric oxide in normotensive and hypertensive rats (Figure 7).

Elevation in blood pressure was accompanied by changes in HDL functions and cholesterol contents. The exact biology and metabolism of HDL in hypertension are, however, not clearly understood. In this study, we examined lipids and lipoprotein metabolism in a rodent model of hypertension (Supplementary Figure S1). Our data suggest that serum lipids are altered in hypertensive rats compared to normotensive controls. We demonstrate reduced total cholesterol and triglycerides and increased free cholesterol, non-HDL-C, and S1P in serum samples from SHRs. Furthermore, HDL composition and subpopulations were altered in SHRs. HDL of SHRs had lower cholesterol levels, higher S1P contents, reduced levels of large HDL particles, and increased levels of medium particles relative to that of WKYs. Reduced total plasma cholesterol, triglycerides, and HDL-C were previously reported in SHRs [24]. The levels of ceramides, a precursor of S1P, were reported to be increased in SHRs and hypertensive individuals. S1P levels, however, were similar between normotensive and hypertensive rodents and humans [25]. The discrepancy between our data and data reported by Spijkers et al. [25] could be due to differences in the rats’ age (3 months vs. 6 months) or the method used to estimate S1P levels (ELISA vs. LC-MS). In agreement with our findings, alterations in HDL composition, function, and properties were reported in hypertensive individuals [7,26]. Higher HDL2 to total HDL cholesterol ratios were associated with reduced risk of incident hypertension [27], while increased HDL3 cholesterol was reported in hypertensive patients [28]. Moreover, antihypertensive medications [29] and exercise [30] reduced HDL3 and increased HDL2 subtractions in hypertensive individuals.

HDL protected hearts of normotensive rodents against I/R injury, recently reviewed in [31]. Our previous [20] and current data suggest that HDL also protects hearts of hypertensive rodents against I/R injury. HDL infusion protected hearts of WKYs and SHRs against I/R injury in a dose-dependent manner (Figure 2). Several lines of evidence have suggested that small dense HDL particles, HDL3, are more protective against CVD than larger particles, reviewed in [32]. Infusion of reconstituted HDL containing apo-AI, apo-AI memetic, or apo-AI Milano protects against myocardial I/R injury in experimental animals [12,33]. We demonstrate that infusion of HDL3 or lipid-free apo-AI after ischemia, ex vivo, is not sufficient to induce myocardial protection in normotensive or hypertensive rats (Supplementary Figure S3), possibly suggesting the requirement of larger HDL particles, apo-AI lipidation, and/or additional HDL components in HDL-mediated cardiac protection. Consistent with our data, Rossoni et al. reported a lack of protective effect of apo A-I against myocardial I/R injury when administered ex vivo before or after the ischemic insult [12]. In addition to HDL administration at reperfusion (posttreatment), administration of HDL prior to ligation or prior to sacrifice (pretreatment) protected WKYs and SHRs against myocardial I/R injury (Supplementary Figure S4). Altogether, these data suggest that acute pretreatment and posttreatment (Supplementary Figure S4) and chronic pretreatment [20] with HDL protect against myocardial I/R injury in SHRs. The compelling findings demonstrating the protective benefits of HDL in both pre- and posttreatment settings offer strong support for the prospective clinical application of HDL in the treatment of myocardial infarction among hypertensive patients.

Consistent with our previous data [20], hearts of normotensive and hypertensive rats responded differently to HDL. HDL (400 µg) induced significantly greater protection in WKYs compared to SHRs (Figure 2). HDL-induced cardiac protection in WKYs and SHRs required HDL binding to SR-BI and blockage of SR-BI, with SR-BI blocking antibody, completely abrogating the response to HDL in normotensive and hypertensive rats [20]. HDL carries bioactive lipids, including S1P, that mediate its protective actions [11,22,34]. In this study, we report the requirement of SR-BI-mediated lipid uptake in HDL-mediated protection against myocardial I/R injury in WKYs and SHRs (Figure 3). Inhibition of SR-BI-mediated lipid uptake revoked the protective effects of HDL in WKYs and SHRs, suggesting its requirement in HDL-induced cardiac protection in normotensive and hypertensive rats. S1P in HDL stimulated the interaction between SR-BI and S1PR1 [22]. S1PR3 mediated the cardioprotective effects of HDL in an in vivo mouse model of myocardial infarction [11]. HDL signaling via S1PR1 and S1PR3 protected cardiomyocytes subjected to hypoxia/reoxygenation [35]. Our data suggest that blockage of S1PR1 and S1PR3 abrogated the protective actions of HDL in WKYs and SHRs. This, however, was not the case for S1PR2. HDL maintained its protective effects in the presence of S1PR2 antagonist (Figure 4). Together, these data suggest that both S1PR1 and S1PR3 are required for HDL-induced cardiac protection in WKYs and SHRs; however, S1PR2 appears to be dispensable. Notably, hearts from WKYs and SHRs exhibited distinct expression of S1PR1 and S1PR3, and intriguingly, S1PR1 and S1PR3 expression displayed contrasting correlations with HDL-mediated augmentation of coronary flow (Supplementary Table S2). Moreover, S1PR3 was more abundantly expressed than S1PR1 in heart membrane fractions in both genotypes (Figure 4J), possibly implicating a principal role of S1PR3 over S1PR1 in cardiac protection. In addition to increased cardiac SR-BI expression [20], elevated cardiac expression of S1PR3 in WKYs (Figure 4I,J), coupled with the observed positive correlation between S1PR3 expression and the HDL-mediated increase in coronary flow (Supplementary Table S2), offers a potential explanation for the enhanced cardiac protection conferred by HDL in WKYs. In contrast to our data, Ahmad et al. reported enhanced S1PR1 expression relative to S1PR3 in rat and human hearts [21]. The discrepancy between this study and our data is not clear. It could, however, be due to differences in the species used (Sprague-Dawley vs. WKYs and SHRs), the technique used to examine receptor expression levels (quantitative real-time PCR; RNA vs. ELISA; protein), or the type of samples used (total homogenates vs. membrane preparations).

We examined the role of mediators of the SAFE and RISK pathways in HDL-induced cardiac protection. HDL-mediated cardiac protection was maintained in the presence of PI3K antagonist but was abolished in the presence of MEK, STAT-3, or JNK1/2 antagonist (Figure 5). This suggests that blockage of PI3K is not sufficient to abrogate the protective effects of HDL in SHR; however, blockage of MEK-ERK1/2, JNK1/2, and STAT-3 signaling appears to be detrimental. This further sheds light on the hierarchy of these signaling pathways, whereby PI3K is likely to act upstream, while downstream targets including Akt, ERK1/2, JNK1/2, and STAT-3 could possibly be activated by PI3K-independent pathway(s). Ischemic pretreatment-induced protection independent of PI3K has recently been reported [36]. Our data are consistent with the reported involvement of Akt and ERK1/2 [35] and STAT-3 [15] in HDL-mediated protection in adult mouse cardiomyocytes. HDL-induced activation of ERK1/2 has been reported to be dependent on S1PR1, while HDL-mediated activation of Akt is dependent on S1PR3 in adult cardiomyocytes [35]. Different components of the RISK pathway interact with each other and with multiple components of the SAFE pathway to mediate cardiac protection [36,37,38]. The nature of this interaction in HDL-mediated cardiac protection in SHRs, however, remains to be investigated. HDL treatment significantly increased Akt and STAT-3 phosphorylation in SHRs and WKYs, respectively. Hearts from SHRs expressed significantly lower levels of total STAT-3, possibly suggesting a reduced response to HDL and/or enhanced susceptibility of these hearts to ischemic damage. However, no changes in the phosphorylation state of ERK1/2 or JNK1/2 were detected in response to HDL treatment in WKYs and SHRs. The observed differences in HDL effects between WKYs and SHRs (Akt and STAT-3) and lack of HDL effect (ERK1/2 and JNK1/2) on the phosphorylation state of these signaling molecules could perhaps be due to differences in the dynamics of HDL-induced signaling between the two genotypes. Differences in the expression levels of SR-BI [20], S1PR1, and S1PR3 (Figure 4) further support this possibility. Enhanced expression of SR-BI and S1PR3 in WKYs may result in a rapid repose to HDL, detection of which could have been missed 30 min after reperfusion.

Activation of mediators of the SAFE and RISK pathways activated cardiac nitric oxide synthase, generated nitric oxide, and protected against cardiac injury [39]. We demonstrate that inhibition of nitric oxide synthase reduced the protective effects of HDL against myocardial I/R injury in normotensive and hypertensive rats (Figure 6A–F, Supplementary Figure S2). Furthermore, we report enhanced generation of nitric oxide, measured as nitrite, in response to HDL treatment in WKYs and SHRs (Figure 6G–J), which correlated positively with enhanced coronary flow in HDL-treated hearts (Supplementary Table S2). Our findings are in line with previous studies reporting that the vasodilatory effects of HDL within coronary circulation are mediated by nitric oxide-dependent mechanisms [11,40]. We further showcase that HDL-mediated nitric oxide generation was completely abolished in the presence of SR-BI blocking antibody and BLT-1, suggesting the requirement of HDL binding to SR-BI and SR-BI-mediated lipid uptake in normotensive and hypertensive rats. In addition, HDL-mediated production of nitric oxide was reduced in the presence of S1PR1 and S1PR3 antagonists (~40% reduction); however, it was abolished in the presence of S1PR2 antagonist (100% reduction). This indicates an essential role of S1PR2 in the HDL-induced generation of nitric oxide that cannot be compensated by S1PR1 or S1PR3. The finding that S1PR2 is not required for HDL-mediated cardiac protection, yet it is crucial for HDL-mediated nitric oxide generation supports the presence of nitric oxide-independent mechanism(s) by which HDL protects against myocardial I/R injury. In addition to meditating the release of nitric oxide [11], HDL-induced protection against myocardial I/R injury involves activation of protective processes including the activation of pro-survival pathways [15] and release of prostanoids [10]. HDL protects against myocardial I/R injury by limiting the diffusion of damaging molecules through gap junction channels via modulating the phosphorylation state of the gap junction protein Cx43 [41]. HDL administration preserves mitochondrial integrity, inhibits mitochondrial permeability transition pore (mPTP) opening, and protects against myocardial I/R injury [15]. Moreover, HDL protects against myocardial I/R injury by inhibiting the release of pro-inflammatory cytokines, chemokines, and reactive oxygen species [10,12] and reducing the expression of microRNAs involved in ischemia reperfusion injury [42]. HDL-induced generation of nitric oxide was reduced in the presence of inhibitors of ERK1/2, STAT-3, or JNK1/2 in hypertensive rats but not normotensive rats, suggesting that blockage of one pathway can be compensated by other pathways in normotensive rats. This complementarity between mediators of the SAFE and RISK pathways appears, however, to be lost in hypertensive rats. The lack of signal input from the PI3K-Akt pathway was effectively compensated by PI3K-independent activation of ERK in remodeled myocardium [38]. This compensatory mechanism enabled erythropoietin-induced protection to be achieved, highlighting the complex interplay between different signaling pathways in maintaining cardiac health and response to therapeutic interventions [38]. Our finding that the compensation between the SAFE and RISK mediators is lost in SHRs suggests a defect in the intricate regulatory mechanisms that allow for crosstalk and/or compensation and implicate impaired response in these rats. Finally, inhibition of L-NAME completely abolished HDL-induced generation of NO in WKYs, yet it reduced it to about 50% in SHRs possibly suggesting the presence of NOS-independent generation of nitrite in SHRs. Increased plasma nitrite and aortic nitrate have been reported in SHRs [43]. Hearts of SHRs expressed increased activity of constitutive nitric oxide synthase, which has been suggested as a compensation against increased blood pressure in these animals [44]. We observed a trend towards an increase in basal nitrite levels in heart homogenates from SHRs relative to WKYs; nonetheless, it did not reach statistical significance. Interestingly, nitric oxide synthase-independent release of nitric oxide has been reported [45]. Nitric oxide reacts with proteins and forms protein-bound dinitrosyl non-heme iron complexes (DNICs). These complexes are stored in non-endothelial cells and contribute to the relaxation of blood vessels [45]. Elevated plasma nitric oxide levels in SHRs are attributed to enhanced release of nitric oxide from DNIC stores in the vasculature [43]. We speculate that the detected nitrite in SHR hearts treated with HDL in the presence of L-NAME (Figure 6J) could perhaps be derived from DNIC stores in these rats. Nonetheless, this possibility remains to be directly investigated. The lack of HDL-mediated cardiac protection in L-NAME-treated SHRs could possibly be due to reduced HDL-induced generation of nitric oxide. Alternatively, it could be due to impaired nitic oxide-mediated signaling in SHRs. Nitric oxide relaxes blood vessels via signaling through guanylyl cyclase and with the concomitant formation of cyclic GMP [46]. Downregulation of guanylyl cyclase has been reported in SHR aortas [47]. HDL antagonizes the inhibitory effect of oxidized low-density lipoprotein and lysolecithin on guanyl cyclase [48]. The effect of HDL on nitric oxide-mediated activation of guanylyl cyclase in hypertension, however, remains to be investigated.

## 5. Conclusions

In conclusion, we demonstrate that the HDL composition and subpopulations were altered in SHRs. HDL pretreatment and posttreatment protected hypertensive rats against myocardial I/R injury. HDL-induced protection was dose-dependent and required SR-BI-mediated lipid uptake. Moreover, HDL required S1PR1 and S1PR3 to protect against I/R injury. HDL-mediated cardiac protection was proportional to the expression levels of S1PR3. Finally, HDL-induced protection involved signaling via Akt, ERK1/2, JNK1/2, and STAT-3 and involved the generation of nitric oxide.

## Data Availability

Data will be available upon request.

## Supplementary Materials

The following supporting information can be downloaded at: https://www.mdpi.com/article/10.3390/pharmaceutics16040497/s1, Figure S1: Serum lipids and lipoprotein analysis in WKY and SHR. Serum samples were isolated from 12-wk old WKY and SHR and assayed enzymatically for total serum cholesterol (A), triglycerides (B), non-HDL cholesterol was calculated by subtracting HDL cholesterol from total cholesterol (C), serum free cholesterol (D), HDL cholesterol (E), HDL-free cholesterol (F) and serum and HDL associated S1P (G). HDL subpopulations were analyzed from the same samples using the lipoprotein system (H). Data is mean ± SEM. * *p* < 0.05, ** *p* < 0.01, **** *p* < 0.001, ns: not significant. Figure S2: Infarct size determination in WKY and SHR. Infarct size in hearts subjected to I/R injury was examined by TTC staining from normotensive (A) and hypertensive rats (B). Data are mean ± SEM of up to five sections per heart. * *p* < 0.05, ** *p* < 0.01 vs. untreated control of the same genotype, # *p* < 0.05, ## *p* < 0.01 vs. HDL (400 µg). Figure S3: HDL induced cardiac protection is not mimicked by lipid free apo-AI or small HDL particle. Post-ischemic recovery parameters of the heart functions including DPmax (A), LVEDP (B), cardiac contractility (D and E), CF (C) and CVR (F). Data were computed at 30 min of HDL (400 µg), apo-AI (400 µg) or HDL3 (100 µg) infused at reperfusion. DPmax, maximum developed pressure; LVEDP, left ventricular end diastolic pressure; CF, coronary flow; CVR, coronary vascular resistance. Data are means ± SEM. *** *p* < 0.001 vs. untreated control; ### *p* < 0.001 vs. ischemia; $$$ *p* < 0.001 vs. SHR+HDL (400 µg). Figure S4: HDL posttreatment and pretreatment protect WKY and SHR against I/R injury. Post-ischemic recovery parameters of the heart functions including DPmax (A), LVEDP (B), cardiac contractility (D and E), CF (C) and CVR (F). HDL (400 µg) was infused prior to reperfusion or prior to ligation or administered intravenously prior to sacrifice. DPmax, maximum developed pressure; LVEDP, left ventricular end diastolic pressure; CF, coronary flow; CVR, coronary vascular resistance. Data are means ± SEM. * *p* < 0.05, ** *p* < 0.01, *** *p* < 0.001 vs. untreated control; # *p* <0.05, ## *p* <0.01, ### *p* <0.001 vs. ischemia; $ *p* < 0.05, $$ *p* < 0.01, $$$ *p* < 0.001 vs. SHR+HDL (400 µg). Supplementary Figure S5: HDL differentially activates SAFE and RISK pro-survival pathways in WKY and SHR. Total heart homogenates from WKY and SHR treated with or without HDL (400 µg) at reperfusion were subjected to immunoblotting against phospho- and total Akt (A), STAT-3 (B and C), ERK 1/2 (E) and JNK1/2 (F) followed by anti-GAPDH as a loading control. Data is mean ± SEM. * *p* < 0.05. Table S1: Blood pressure, body weight and heart weight of WKY and SHR. Table S2: Relationship between coronary flow and SRR-BI, S1PRs and nitrite levels in HDL treated hearts from WKY and SHR. * *p* < 0.05, ** *p* < 0.001.

## References

[1] Lawes C.M., Vander Hoorn S., Rodgers A., International Society of Hypertension (2008). Global burden of blood-pressure-related disease, 2001. Lancet.

[2] Yusuf S., Sleight P., Pogue J., Bosch J., Davies R., Dagenais G. (2000). Effects of an angiotensin-converting-enzyme inhibitor, ramipril, on cardiovascular events in high-risk patients. The Heart Outcomes Prevention Evaluation Study Investigators. N. Engl. J. Med..

[3] Hamad A., Salameh M., Mahmoud H., Singh J., Zaghmout M., Ward L. (2001). Relation of high levels of high-density lipoprotein cholesterol to coronary artery disease and systemic hypertension. Am. J. Cardiol..

[4] Castelli W.P., Garrison R.J., Wilson P.W., Abbott R.D., Kalousdian S., Kannel W.B. (1986). Incidence of coronary heart disease and lipoprotein cholesterol levels. The Framingham Study. JAMA.

[5] Oyama N., Sakuma I., Kishimoto N., Saijo Y., Sakai H., Urasawa K., Kitabatake A., Kishi R., Tsutsui H. (2006). Low HDL-cholesterol, hypertension and impaired glucose tolerance as predictors of acute myocardial infarction in northern area of Japan. Hokkaido Igaku Zasshi.

[6] Al-Jarallah A., Trigatti B.L. (2010). A role for the scavenger receptor, class B type I in high density lipoprotein dependent activation of cellular signaling pathways. Biochim. Biophys. Acta.

[7] Ozerova I.N., Perova N.V., Shchel’tsyna N.V., Mamedov M.N. (2007). Parameters of high-density lipoproteins in patients with arterial hypertension in combination with other components of metabolic syndrome. Bull. Exp. Biol. Med..

[8] Einbinder Y., Biron-Shental T., Agassi-Zaitler M., Tzadikevitch-Geffen K., Vaya J., Khatib S., Ohana M., Benchetrit S., Zitman-Gal T. (2018). High-density lipoproteins (HDL) composition and function in preeclampsia. Arch. Gynecol. Obstet..

[9] Hansel B., Girerd X., Bonnefont-Rousselot D., Bittar R., Chantepie S., Orsoni A., Bruckert E., Chapman M.J., Kontush A. (2011). Blood pressure-lowering response to amlodipine as a determinant of the antioxidative activity of small, dense HDL3. Am. J. Cardiovasc. Drugs.

[10] Calabresi L., Rossoni G., Gomaraschi M., Sisto F., Berti F., Franceschini G. (2003). High-density lipoproteins protect isolated rat hearts from ischemia-reperfusion injury by reducing cardiac tumor necrosis factor-alpha content and enhancing prostaglandin release. Circ. Res..

[11] Theilmeier G., Schmidt C., Herrmann J., Keul P., Schafers M., Herrgott I., Mersmann J., Larmann J., Hermann S., Stypmann J. (2006). High-density lipoproteins and their constituent, sphingosine-1-phosphate, directly protect the heart against ischemia/reperfusion injury in vivo via the S1P3 lysophospholipid receptor. Circulation.

[12] Rossoni G., Gomaraschi M., Berti F., Sirtori C.R., Franceschini G., Calabresi L. (2004). Synthetic high-density lipoproteins exert cardioprotective effects in myocardial ischemia/reperfusion injury. J. Pharmacol. Exp. Ther..

[13] Marie-Claude B.-M., Vincent B., Jonas W.B., Jonas B., Fabrizio M., Jean-Christophe P., Jean-Christophe P., Aurélien T., Katia G., Graziano P. (2015). Improving Reconstituted HDL Composition for Efficient Post-Ischemic Reduction of Ischemia Reperfusion Injury. PLoS ONE.

[14] Frangogiannis N.G., Lindsey M.L., Michael L.H., Youker K.A., Bressler R.B., Mendoza L.H., Spengler R.N., Smith C.W., Entman M.L. (1998). Resident cardiac mast cells degranulate and release preformed TNF-alpha, initiating the cytokine cascade in experimental canine myocardial ischemia/reperfusion. Circulation.

[15] Frias M.A., Pedretti S., Hacking D., Somers S., Lacerda L., Opie L.H., James R.W., Lecour S. (2013). HDL protects against ischemia reperfusion injury by preserving mitochondrial integrity. Atherosclerosis.

[16] Wagner C., Ebner B., Tillack D., Strasser R.H., Weinbrenner C. (2013). Cardioprotection by ischemic postconditioning is abrogated in hypertrophied myocardium of spontaneously hypertensive rats. J. Cardiovasc. Pharmacol..

[17] Yano T., Miki T., Tanno M., Kuno A., Itoh T., Takada A., Sato T., Kouzu H., Shimamoto K., Miura T. (2011). Hypertensive hypertrophied myocardium is vulnerable to infarction and refractory to erythropoietin-induced protection. Hypertension.

[18] Oei G.T., Huhn R., Heinen A., Hollmann M.W., Schlack W.S., Preckel B., Weber N.C. (2012). Helium-induced cardioprotection of healthy and hypertensive rat myocardium in vivo. Eur. J. Pharmacol..

[19] Penna C., Tullio F., Moro F., Folino A., Merlino A., Pagliaro P. (2010). Effects of a protocol of ischemic postconditioning and/or captopril in hearts of normotensive and hypertensive rats. Basic. Res. Cardiol..

[20] Al-Jarallah A., Babiker F. (2022). High Density Lipoprotein Reduces Blood Pressure and Protects Spontaneously Hypertensive Rats Against Myocardial Ischemia-Reperfusion Injury in an SR-BI Dependent Manner. Front. Cardiovasc. Med..

[21] Ahmed N., Linardi D., Decimo I., Mehboob R., Gebrie M.A., Innamorati G., Luciani G.B., Faggian G., Rungatscher A. (2017). Characterization and Expression of Sphingosine 1-Phosphate Receptors in Human and Rat Heart. Front. Pharmacol..

[22] Lee M.H., Appleton K.M., El-Shewy H.M., Sorci-Thomas M.G., Thomas M.J., Lopes-Virella M.F., Luttrell L.M., Hammad S.M., Klein R.L. (2017). S1P in HDL promotes interaction between SR-BI and S1PR1 and activates S1PR1-mediated biological functions: Calcium flux and S1PR1 internalization. J. Lipid Res..

[23] Nofer J.R. (2015). Signal transduction by HDL: Agonists, receptors, and signaling cascades. Handb. Exp. Pharmacol..

[24] Kitts D.D., Yuan Y.V., Godin D.V. (1998). Plasma and lipoprotein lipid composition and hepatic antioxidant status in spontaneously hypertensive (SHR) and normotensive (WKY) rats. Can. J. Physiol. Pharmacol..

[25] Spijkers L.J., van den Akker R.F., Janssen B.J., Debets J.J., De Mey J.G., Stroes E.S., van den Born B.J., Wijesinghe D.S., Chalfant C.E., MacAleese L. (2011). Hypertension is associated with marked alterations in sphingolipid biology: A potential role for ceramide. PLoS ONE.

[26] Sheu W.H., Swislocki A.L., Hoffman B.B., Reaven G.M., Chen Y.D. (1990). Effect of prazosin treatment on HDL kinetics in patients with hypertension. Am. J. Hypertens..

[27] You-Cheol H., Wilfred Y.F., Steven E.K., Donna L.L., Edward J.B. (2019). Higher High Density Lipoprotein 2 (HDL2) to Total HDL Cholesterol Ratio Is Associated with a Lower Risk for Incident Hypertension. Diabetes Metab. J..

[28] Absetou G., Koumaré A.T.C.R.K., Talkmore M., Samandoulougou A., Kabré E., Sondé I., Simporé J., Sakandé J. (2015). Plasma lipid profile including the high density lipoprotein (HDL) subclasses in hypertensive patients in Ouagadougou, Burkina Faso. Afr. J. Biochem. Res..

[29] Sasaki J., Jun S., Arakawa K. (1989). Effect of captopril on high-density lipoprotein subfractions in patients with mild to moderate essential hypertension. Clin. Ther..

[30] Nieto O.A., Garcia D.M., Jimenez J.A., Landazuri P. (2013). [Effect of exercise on high density lipoprotein subpopulations and blood pressure]. Rev. Salud. Publica.

[31] Sposito A.C., de Lima J.C., Moura F.A., Barreto J., Bonilha I., Santana M., Virginio V.W., Sun L., Carvalho L.S.F., Soares A.A.S. (2019). Reciprocal Multifaceted Interaction Between HDL (High-Density Lipoprotein) and Myocardial Infarction. Arterioscler. Thromb. Vasc. Biol..

[32] Woudberg N.J., Pedretti S., Lecour S., Schulz R., Vuilleumier N., James R.W., Frias M.A. (2017). Pharmacological Intervention to Modulate HDL: What Do We Target?. Front. Pharmacol..

[33] Marchesi M., Booth E.A., Davis T., Bisgaier C.L., Lucchesi B.R. (2004). Apolipoprotein A-IMilano and 1-palmitoyl-2-oleoyl phosphatidylcholine complex (ETC-216) protects the in vivo rabbit heart from regional ischemia-reperfusion injury. J. Pharmacol. Exp. Ther..

[34] Elena M.G.D.-A., Alejandra M.-L.K., Antonio P., Antonio P., Núria A., Núria A., Francisco B.-V., Josep J. (2019). Novel Insights into the Role of HDL-Associated Sphingosine-1-Phosphate in Cardiometabolic Diseases. Int. J. Mol. Sci..

[35] Tao R., Hoover H.E., Honbo N., Kalinowski M., Alano C.C., Karliner J.S., Raffai R. (2010). High-density lipoprotein determines adult mouse cardiomyocyte fate after hypoxia-reoxygenation through lipoprotein-associated sphingosine 1-phosphate. American journal of physiology. Heart Circ. Physiol..

[36] Rossello X., Riquelme J.A., Davidson S.M., Yellon D.M. (2018). Role of PI3K in myocardial ischaemic preconditioning: Mapping pro-survival cascades at the trigger phase and at reperfusion. J. Cell Mol. Med..

[37] Frias M.A., James R.W., Gerber-Wicht C., Lang U. (2009). Native and reconstituted HDL activate Stat3 in ventricular cardiomyocytes via ERK1/2: Role of sphingosine-1-phosphate. Cardiovasc. Res..

[38] Miki T., Miura T., Tanno M., Nishihara M., Naitoh K., Sato T., Takahashi A., Shimamoto K. (2007). Impairment of cardioprotective PI3K-Akt signaling by post-infarct ventricular remodeling is compensated by an ERK-mediated pathway. Basic. Res. Cardiol..

[39] Xia Z., Vanhoutte P.M. (2011). Nitric oxide and protection against cardiac ischemia. Curr. Pharm. Des..

[40] Levkau B., Hermann S., Theilmeier G., van der Giet M., Chun J., Schober O., Schafers M. (2004). High-density lipoprotein stimulates myocardial perfusion in vivo. Circulation.

[41] Morel S., Frias M.A., Rosker C., James R.W., Rohr S., Kwak B.R. (2012). The natural cardioprotective particle HDL modulates connexin43 gap junction channels. Cardiovasc. Res..

[42] Yung B.S., Brand C.S., Xiang S.Y., Gray C.B., Means C.K., Rosen H., Chun J., Purcell N.H., Brown J.H., Miyamoto S. (2017). Selective coupling of the S1P(3) receptor subtype to S1P-mediated RhoA activation and cardioprotection. J. Mol. Cell Cardiol..

[43] Wu C.C., Yen M.H. (1999). Higher level of plasma nitric oxide in spontaneously hypertensive rats. Am. J. Hypertens..

[44] Nava E., Noll G., Luscher T.F. (1995). Increased activity of constitutive nitric oxide synthase in cardiac endothelium in spontaneous hypertension. Circulation.

[45] Muller B., Kleschyov A.L., Stoclet J.C. (1996). Evidence for N-acetylcysteine-sensitive nitric oxide storage as dinitrosyl-iron complexes in lipopolysaccharide-treated rat aorta. Br. J. Pharmacol..

[46] Surmeli N.B., Muskens F.M., Marletta M.A. (2015). The Influence of Nitric Oxide on Soluble Guanylate Cyclase Regulation by Nucleotides: Role of the Pseudosymmetric Site. J. Biol. Chem..

[47] Ruetten H., Zabel U., Linz W., Schmidt H.H. (1999). Downregulation of soluble guanylyl cyclase in young and aging spontaneously hypertensive rats. Circ. Res..

[48] Schmidt K., Klatt P., Graier W.F., Kostner G.M., Kukovetz W.R. (1992). High-density lipoprotein antagonizes the inhibitory effects of oxidized low-density lipoprotein and lysolecithin on soluble guanylyl cyclase. Biochem. Biophys. Res. Commun..

