# Supplementary material for: High-Density Lipoprotein Signaling via Sphingosine-1-Phosphate Receptors Safeguards Spontaneously Hypertensive Rats against Myocardial Ischemia/Reperfusion Injury
Source: Pharmaceutics. 2024 Apr 3;16(4):497. doi: 10.3390/pharmaceutics16040497 (PMC11054943; doi:10.3390/pharmaceutics16040497)
Supplement: Supplementary file 1 [file pharmaceutics-16-00497-s001.zip › Supplementary Figures.pdf]

Supplementary Figures

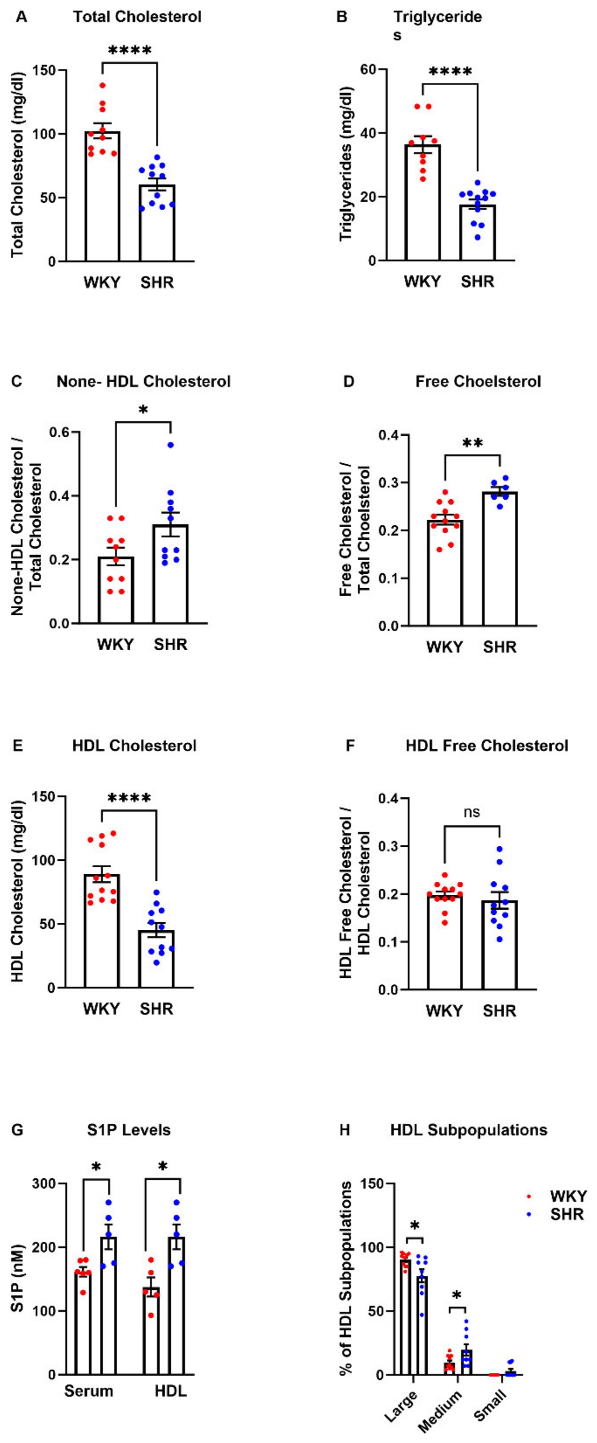

Supplementary Figure S1: Serum lipids and lipoprotein analysis in WKY and SHR. Serum samples were isolated from 12-wk old WKY and SHR and assayed enzymatically for total serum cholesterol (A), triglycerides (B), non-HDL cholesterol was calculated by subtracting HDL cholesterol from total cholesterol (C), serum free cholesterol (D), HDL cholesterol (E), HDL-free cholesterol (F) and serum and HDL associated S1P (G). HDL subpopulations were

analyzed from the same samples using the lipoprotein system (H). Data is mean ± SEM. *P<0.05, **P<0.01, ****P<0.001, ns: not significant.

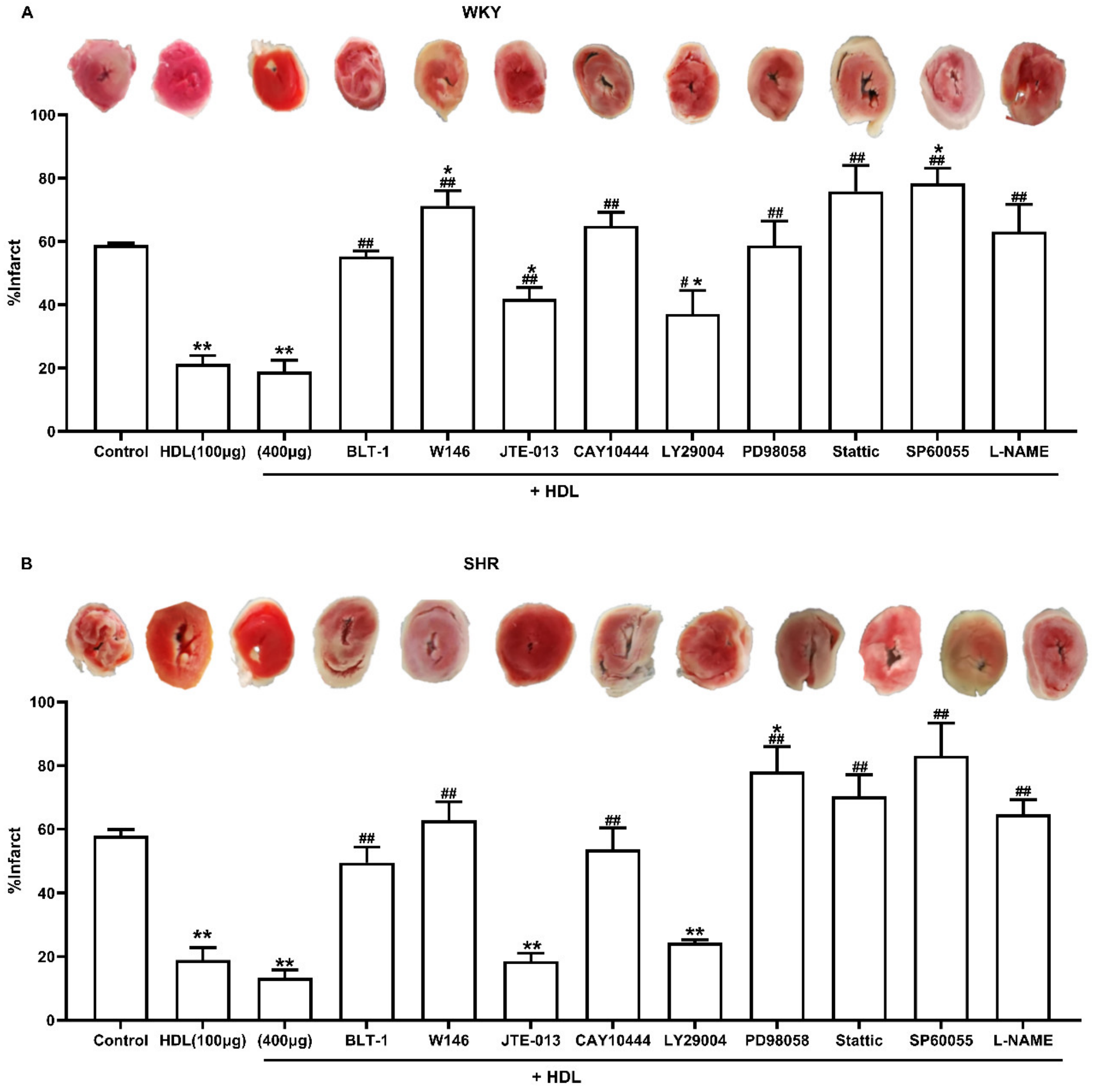

Supplementary Figure S2. Infarct size determination in WKY and SHR. Infarct size in hearts subjected to I/R injury was examined by TTC staining from normotensive (A) and hypertensive rats (B). Data are mean ± SEM of up to five sections per heart. * P<0.05, ** P<0.01 vs. untreated control of the same genotype, # P<0.05, ## P<0.01 vs HDL (400µg).

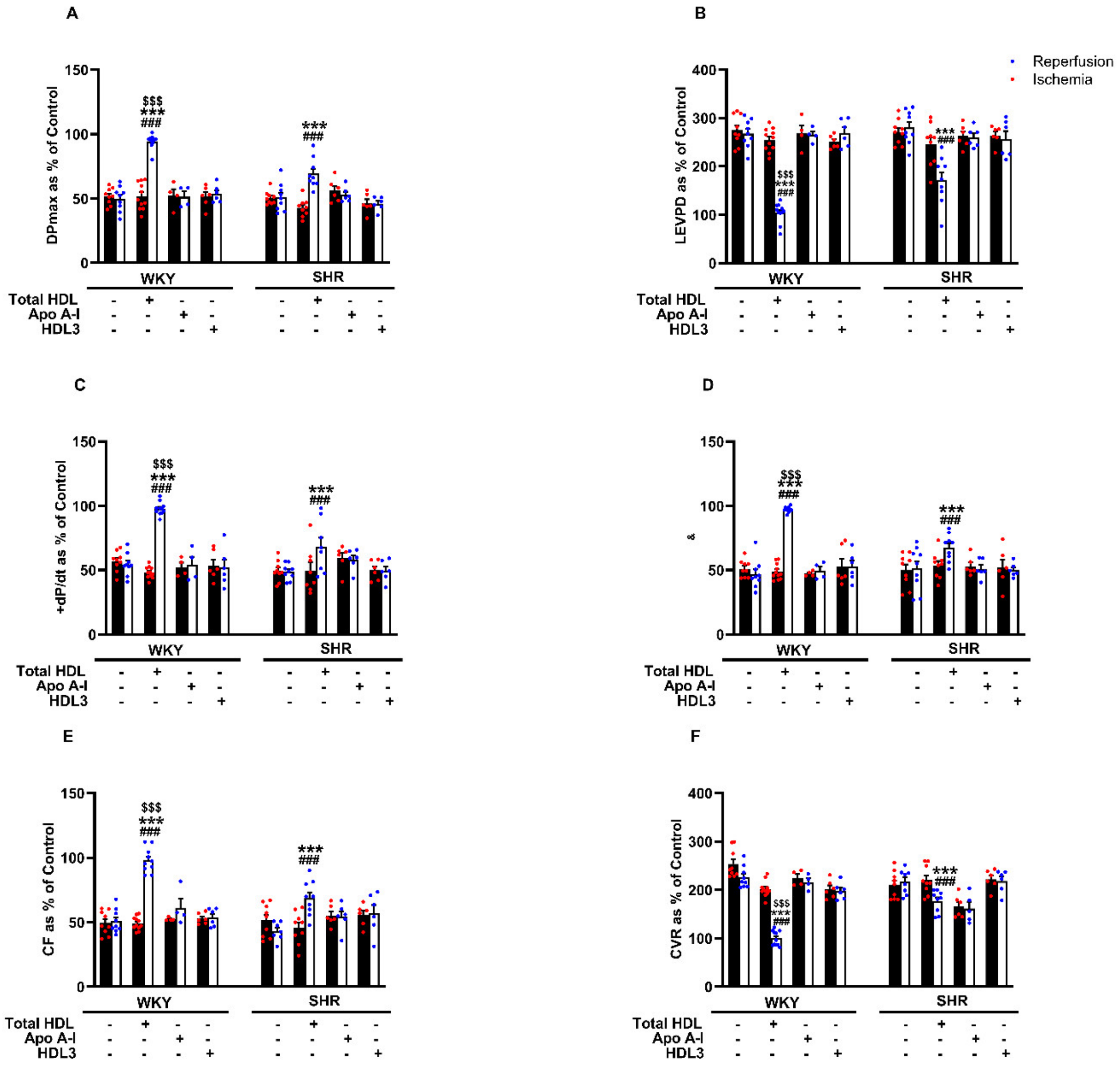

Supplementary Figure S3. HDL induced cardiac protection is not mimicked by lipid free apo-AI or small HDL particle. Post-ischemic recovery parameters of the heart functions including DPmax (A), LVEDP (B), cardiac contractility (D and E), CF (C) and CVR (F). Data were computed at 30 min of HDL (400 µg), apo-AI (400 µg) or HDL3 (100 µg) infused at reperfusion. DPmax, maximum developed pressure; LVEDP, left ventricular end diastolic pressure; CF, coronary flow; CVR, coronary vascular resistance. Data are means ± SEM. ***P<0.001 vs untreated control; $^{###}$P <0.001 vs ischemia; $^{\$ \$\$\$}$P <0.001 vs SHR+HDL (400 µg).

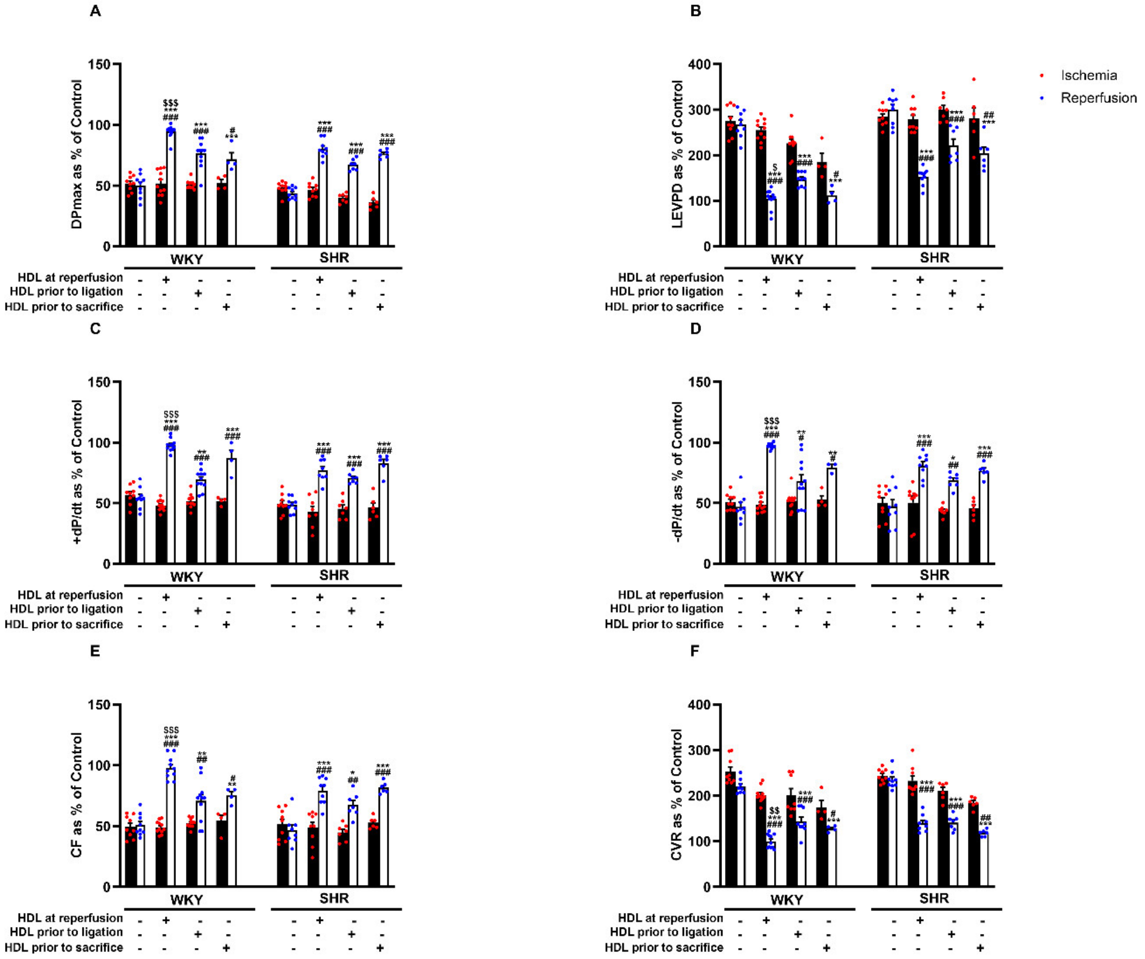

Supplementary Figure S4. HDL posttreatment and pretreatment protect WKY and SHR against I/R injury. Post-ischemic recovery parameters of the heart functions including DPmax (A), LVEDP (B), cardiac contractility (D and E), CF (C) and CVR (F). HDL (400 µg) was infused prior to reperfusion or prior to ligation or administered intravenously prior to sacrifice. DPmax, maximum developed pressure; LVEDP, left ventricular end diastolic pressure; CF, coronary flow; CVR, coronary vascular resistance. Data are means ± SEM. *P < 0.05, **P <0.01, ***P<0.001 vs untreated control; $^{\#}$P <0.05, $^{\#\#}$P <0.01, $^{\#\#\#}$P <0.001 vs ischemia; $^{\$}$P <0.05, $^{\$\$}$P< 0.01, $^{\$\$\$}$P <0.001 vs SHR+HDL (400 µg).

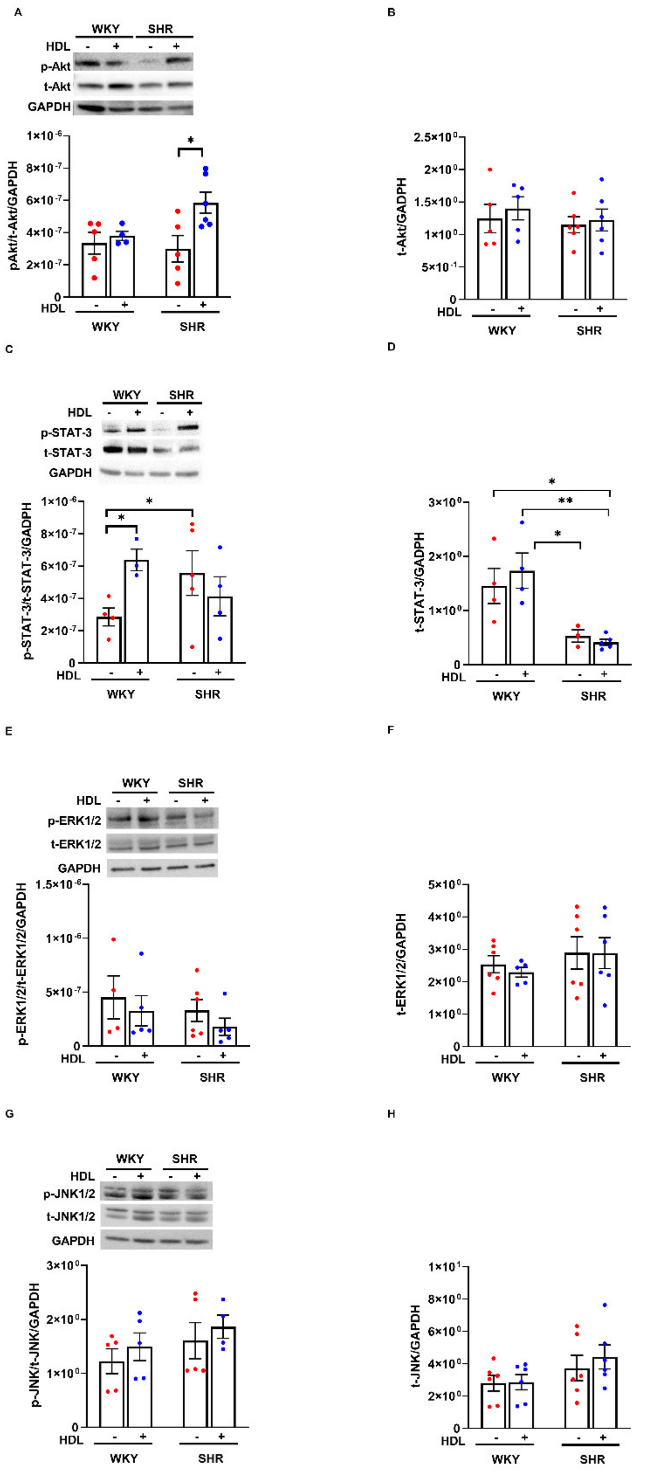

Supplementary Figure S5. HDL differentially activates SAFE and RISK pro-survival pathways in WKY and SHR. Total heart homogenates from WKY and SHR treated with or without HDL (400 μg) at reperfusion were subjected to immunoblotting against phospho- and total Akt (A), STAT-3 (B and C), ERK 1/2 (E) and JNK1/2 (F) followed by anti-GAPDH as a loading control. Data is mean ± SEM. *P < 0.05.
