# Supplementary material for: High-Density Lipoprotein Signaling via Sphingosine-1-Phosphate Receptors Safeguards Spontaneously Hypertensive Rats against Myocardial Ischemia/Reperfusion Injury
Source: Pharmaceutics. 2024 Apr 3;16(4):497. doi: 10.3390/pharmaceutics16040497 (PMC11054943; doi:10.3390/pharmaceutics16040497)
Supplement: Supplementary file 1 [file pharmaceutics-16-00497-s001.zip › Supplementary Tables.pptx]

## Slide 1
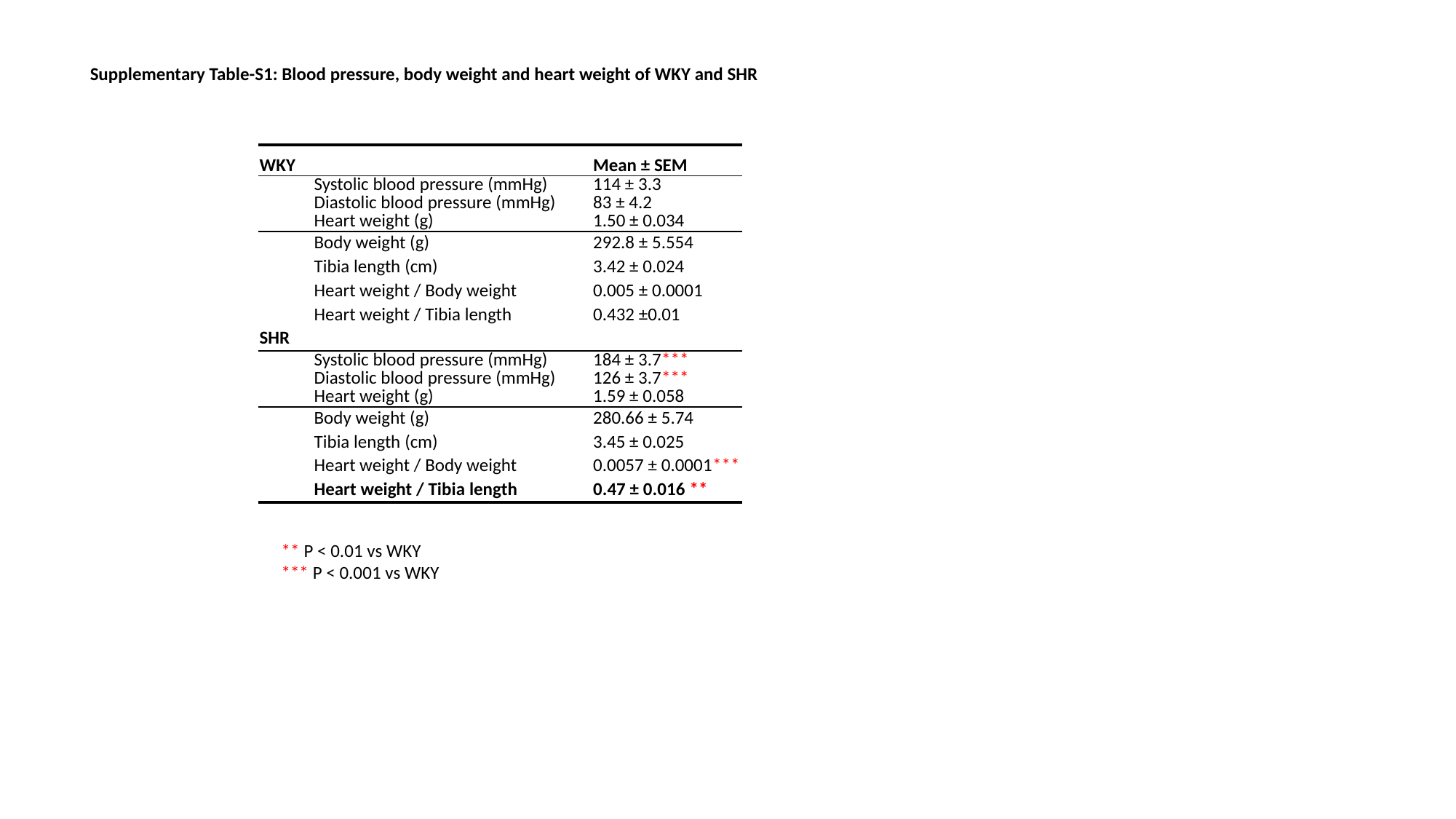

Supplementary Table-S1: Blood pressure, body weight and heart weight of WKY and SHR
| WKY | Mean ± SEM |
| --- | --- |
| Systolic blood pressure (mmHg) Diastolic blood pressure (mmHg) Heart weight (g) | 114 ± 3.3 83 ± 4.2 1.50 ± 0.034 |
| Body weight (g) | 292.8 ± 5.554 |
| Tibia length (cm) | 3.42 ± 0.024 |
| Heart weight / Body weight | 0.005 ± 0.0001 |
| Heart weight / Tibia length | 0.432 ±0.01 |
| SHR | |
| Systolic blood pressure (mmHg) Diastolic blood pressure (mmHg) Heart weight (g) | 184 ± 3.7\*\*\* 126 ± 3.7\*\*\* 1.59 ± 0.058 |
| Body weight (g) | 280.66 ± 5.74 |
| Tibia length (cm) | 3.45 ± 0.025 |
| Heart weight / Body weight | 0.0057 ± 0.0001\*\*\* |
| Heart weight / Tibia length | 0.47 ± 0.016 \*\* |
** P < 0.01 vs WKY
*** P < 0.001 vs WKY

## Slide 2
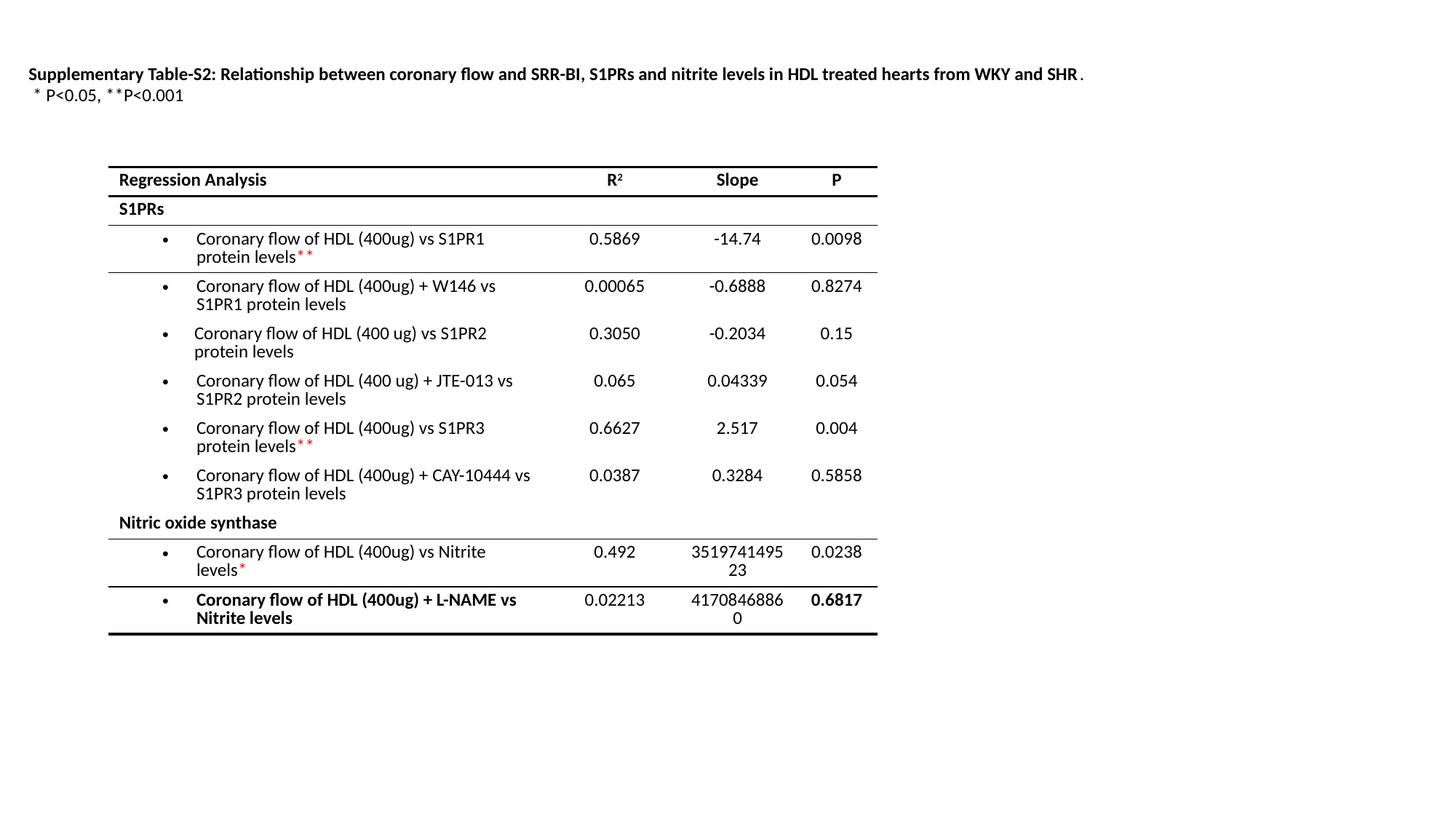

Supplementary Table-S2: Relationship between coronary flow and SRR-BI, S1PRs and nitrite levels in HDL treated hearts from WKY and SHR.
 * P<0.05, **P<0.001
| Regression Analysis | R2 | Slope | P |
| --- | --- | --- | --- |
| S1PRs | | | |
| Coronary flow of HDL (400ug) vs S1PR1 protein levels\*\* | 0.5869 | -14.74 | 0.0098 |
| Coronary flow of HDL (400ug) + W146 vs S1PR1 protein levels | 0.00065 | -0.6888 | 0.8274 |
| Coronary flow of HDL (400 ug) vs S1PR2 protein levels | 0.3050 | -0.2034 | 0.15 |
| Coronary flow of HDL (400 ug) + JTE-013 vs S1PR2 protein levels | 0.065 | 0.04339 | 0.054 |
| Coronary flow of HDL (400ug) vs S1PR3 protein levels\*\* | 0.6627 | 2.517 | 0.004 |
| Coronary flow of HDL (400ug) + CAY-10444 vs S1PR3 protein levels | 0.0387 | 0.3284 | 0.5858 |
| Nitric oxide synthase | | | |
| Coronary flow of HDL (400ug) vs Nitrite levels\* | 0.492 | 351974149523 | 0.0238 |
| Coronary flow of HDL (400ug) + L-NAME vs Nitrite levels | 0.02213 | 41708468860 | 0.6817 |
